# Application of the adverse outcome pathway concept for investigating developmental neurotoxicity potential of Chinese herbal medicines by using human neural progenitor cells in vitro

**DOI:** 10.1007/s10565-022-09730-4

**Published:** 2022-06-15

**Authors:** Jördis Klose, Lu Li, Melanie Pahl, Farina Bendt, Ulrike Hübenthal, Christian Jüngst, Patrick Petzsch, Astrid Schauss, Karl Köhrer, Ping Chung Leung, Chi Chiu Wang, Katharina Koch, Julia Tigges, Xiaohui Fan, Ellen Fritsche

**Affiliations:** 1 IUF – Leibniz-Research Institute for Environmental Medicine, NRW, Auf’m Hennekamp 50, 40225 Duesseldorf, Germany; 2 College of Pharmaceutical Sciences, Zhejiang University, Hangzhou, China; 3 Department of Obstetrics & Gynaecology, School of Biomedical Sciences, Li Ka Shing Institute of Health Sciences, The Chinese University of Hong Kong, Shatin, N.T Hong Kong; 4 Institute of Chinese Medicine, The Chinese University of Hong Kong, Shatin, N.T Hong Kong; 5 State Key Laboratory of Component-Based Chinese Medicine, Innovation Center in Zhejiang University, Hangzhou, China; 6 CECAD Imaging Facility, CECAD Forschungszentrum Cologne, NRW, Joseph-Stelzmann-Str. 26, 50931 Cologne, Germany; 7 Biological and Medical Research Centre (BMFZ), Medical Faculty, Heinrich-Heine-University, NRW, Universitätsstraße 1, 40225 Duesseldorf, Germany; 8 Joint Laboratory in Reproductive Medicine, The Chinese University of Hong Kong and Sichuan University, Hong Kong, China; 9 College of Basic Medical Sciences, Zhejiang Chinese Medical University, Hangzhou, China; 10 Medical Faculty, Heinrich-Heine-University, NRW, Universitätsstraße 1, 40225 Duesseldorf, Germany

**Keywords:** Developmental neurotoxicity, Human neural progenitor cells in vitro, 3D neurosphere assay, Chinese herbal medicines, Adverse outcome pathways, New approach methodologies

## Abstract

Adverse outcome pathways (AOPs) are organized sequences of key events (KEs) that are triggered by a xenobiotic-induced molecular initiating event (MIE) and summit in an adverse outcome (AO) relevant to human or ecological health. The AOP framework causally connects toxicological mechanistic information with apical endpoints for application in regulatory sciences. AOPs are very useful to link endophenotypic, cellular endpoints in vitro to adverse health effects in vivo. In the field of in vitro developmental neurotoxicity (DNT), such cellular endpoints can be assessed using the human “Neurosphere Assay,” which depicts different endophenotypes for a broad variety of neurodevelopmental KEs. Combining this model with large-scale transcriptomics, we evaluated DNT hazards of two selected Chinese herbal medicines (CHMs) Lei Gong Teng (LGT) and Tian Ma (TM), and provided further insight into their modes-of-action (MoA). LGT disrupted hNPC migration eliciting an exceptional migration endophenotype. Time-lapse microscopy and intervention studies indicated that LGT disturbs laminin-dependent cell adhesion. TM impaired oligodendrocyte differentiation in human but not rat NPCs and activated a gene expression network related to oxidative stress. The LGT results supported a previously published AOP on radial glia cell adhesion due to interference with integrin-laminin binding, while the results of TM exposure were incorporated into a novel putative, stressor-based AOP. This study demonstrates that the combination of phenotypic and transcriptomic analyses is a powerful tool to elucidate compounds’ MoA and incorporate the results into novel or existing AOPs for a better perception of the DNT hazard in a regulatory context.

**Graphical abstract:**

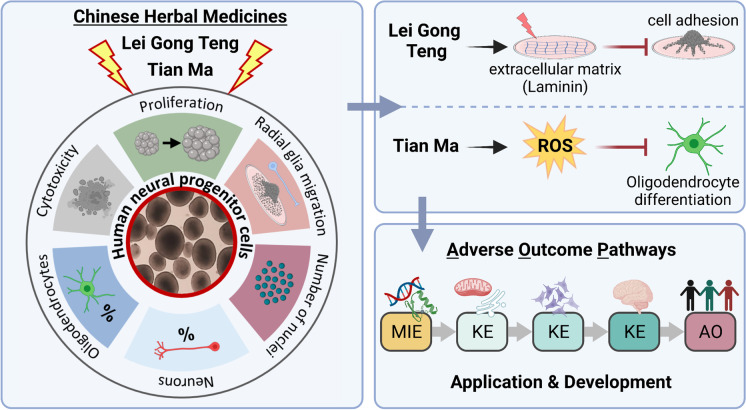

**Supplementary Information:**

The online version contains supplementary material available at 10.1007/s10565-022-09730-4.

## Highlights

- A 3D in vitro model based on human neural progenitor cells was applied for assessing DNT potential of two selected CHMs.
- Both CHMs caused DNT endophenotypes in vitro, e.g. disturbed radial glia migration or impaired oligodendrocyte differentiation.
- Combining phenotypic with transcriptomic analyses enlight compounds’ modes-of-action.
- Application and development of AOPs for a better comprehension of DNT hazards.

**Supplementary Information:**

The online version contains supplementary material available at 10.1007/s10565-022-09730-4.

## Introduction

New approach methodologies (NAMs) are non-animal-based methods, including in vitro approaches, which provide toxicodynamic information on chemical hazards, thereby supporting the proposed paradigm shift in toxicology—moving from the sole use of apical endpoints generated in animals towards a mechanistic understanding and more human-relevant approaches for regulatory applications (NRC 2007; Collins et al. 2008). NAMs are no stand-alone methodologies, yet need embedding into larger frameworks, i.e., integrated approaches for testing and assessment (IATAs). The IATA concept has been proposed by the Organization for Economic Cooperation and Development (OECD) member countries to embed alternative testing strategies into weight of evidence assessment for decision-making using data from various information sources (OECD 2020). IATA information on toxicodynamics might arise from adverse outcome pathways (AOP), which are structured organizations of causally related biological events leading to adverse effects and provide mechanistic information on the molecular initiating event (MIE); molecular, cellular, structural, and functional key events (KEs); and associated key event relationships (KER; Ankley et al. 2010; OECD 2016). Thereby, AOPs serve as a knowledge assembly and communication tool between research and regulatory communities involved, e.g., in regulatory and systems toxicology, biomedical challenges, safety evaluations associated with drug development, and clinical trial simulations (Carusi et al. 2018), thus covering a broad biomedical application domain. An essential platform for AOP documentation, which is currently most developed, is the AOP-Wiki (https://aopwiki.org/), which provides a formalized, transparent, and quality-controlled data documentation and hence facilitates knowledge exchange between different stakeholders (Kandel et al. 2000; Vinken et al. 2017).

Spatiotemporal orchestration of molecular and cellular processes is essential for proper human brain development and contributes to the formation of a functional central nervous system (Silbereis et al. 2016). This complex interplay of highly dynamic neurodevelopmental processes causes a higher vulnerability towards adverse chemical effects of the developing compared to the adult brain (Rodier 1994; Rice et al. 2000). Due to the manifold KEs that happen in a time- and brain region-specific manner, creation of AOPs for developmental neurotoxicity (DNT) that cover the DNT toxicological space will be a continuous effort that is far from being completed at the moment. To date, seven AOPs are endorsed by the OECD, eleven were submitted to the AOP-Wiki (https://aopwiki.org/), and some were published in the scientific literature (Bal-Price and Meek 2017; Barenys et al. 2019; Li et al. 2019a; Spinu et al. 2019; Chen et al. 2020; Klose et al. 2021b). Due to this low abundance of DNT-AOPs, compound hazard characterization for DNT solely on the basis of AOPs using NAMs is very difficult. DNT-NAMs, in contrast, have been contributing to AOP building (Bal-Price et al. 2016; Masjosthusmann et al. 2020; EFSA 2021; Klose et al. 2021b) creating an interesting ongoing interplay in the DNT-AOP arena.

A multitude of Chinese herbal medicines (CHMs) are insufficiently characterized concerning their potential to cause DNT. For centuries, CHMs have been widely used during pregnancy to relieve symptoms like morning sickness (Flaws 2005) and treat pregnancy complications, especially to prevent miscarriage during early gestation (Li et al. 2013). Generally, CHMs are considered safe by the consumers due to their natural origin and availability as teas (Terzioglu Bebitoglu 2020). Yet it cannot be excluded that CHMs may pose adverse effects to the developing child. We recently reported on the plant-derived substance class of flavonoids as compounds with suspected developmental toxicity where—similar to CHMs—the neurodevelopmental potential is so far understudied (Barenys et al. 2017b). We also identified a DNT hazard mode-of-action (MoA) for the green tea catechin epigallocatechin gallate (EGCG; Barenys et al. 2017a).

In the present study, we performed a case study with two selected CHMs, Lei Gong Teng (LGT; *Radix Et Rhizoma Tripterygii Wilfordii*, Common Threewingnut Root) and Tian Ma (TM; *Gastrodia elata Blume*, Tall Gastrodia Tuber) by evaluating their adverse neurodevelopmental effects with the test methods NPC1-5 (Koch et al. 2022), which are based on developing human neural progenitor cells (hNPCs) in the “Neurosphere Assay,” which is part of a current OECD/EFSA (European Food Safety Authority) DNT in vitro battery (IVB; Masjosthusmann et al. 2020; EFSA 2021). Data generated in this study are placed in an AOP context by applying one and expanding a second already existing putative AOP for DNT.

## Materials and methods

### Chemicals

LGT and TM were purchased, quality-controlled, prepared, and extracted from crude herbs into powders by an authorized lab within the Institute of Chinese Medicine at the Chinese University of Hong Kong under the supervision of Prof. Ping Chung Leung. The chemical authentication was conducted by thin-layer chromatography (TLC) or high-performance liquid chromatography (HPLC) according to guidelines of the Chinese Pharmacopoeia (Chinese Pharmacopoeia Commission 2020). This confirmed the quality and quantity of chemical components in LGT and TM, also excluding any pesticide, mineral, and other biological contamination. The preparation process of the crude herbs included cleaning, washing, cutting, grinding, and homogenization into fragments. In the extraction process, LGT and TM were separately decocted with 250 mL boiling distilled water at 100 °C for 2 h. The decoction (25 mL) was filtered, concentrated, and dried into powdered form using a spray-dryer and kept in a desiccator prior to use. Each extraction was prepared in one batch, and the yield rate was 6.55% and 65.44% for LGT and TM, respectively. Therefore, both crude CHMs are purified mixtures and the ingredients included are listed in supplementary Table S2, which are based on records in the Chinese Pharmacopoeia and/or reports in literatures (Chinese Pharmacopoeia Commission 2020).

Both powders were diluted directly in cell culture medium and 1 mg/mL (LGT) and 2 mg/mL (TM) stock solutions were prepared and stored at 4 °C for a maximum of 2 days. These stock concentrations are the respective solubility limits for both powders used in this study. Epigallocatechin gallate (EGCG; > 98%) was purchased from TransMIT PlantMetaChem (Giessen, Germany). A stock solution of 10 mM in DMSO was prepared and stored at − 20 °C. The final solvent concentration for EGCG was 0.1% DMSO in microarray experiments.

### Neurosphere cell culture

hNPCs were isolated from cortices of gestational week (GW) 16–19 fetuses and purchased from Lonza (Verviers SPRL, Belgium (#PT-2599)). For this project, three different male individuals (Lot No.: 0000391398 (GW19), 0,000,549,062 (GW16), 0,000,516,385 (GW16)) were used. hNPCs were thawed and cultured as previously described (Baumann et al. 2014; Nimtz et al. 2019). Time-matched rat NPCs (rNPCs) were isolated from postnatal day 1 (PND 1) pubs by dissecting, digesting, and homogenizing whole brains to obtain a cell suspension that spontaneously formed free-floating neurospheres (Baumann et al. 2014). The preparation of the rat pubs was approved by the “Landesamt für Natur, Umwelt und Verbraucherschutz” (LANUV; 81–02.05.50.18.001) and performed according to law on animal welfare §4 Abs. 3 Tierschutzgesetzt (TierSchG). NPCs were cultured as 3D free-floating neurospheres in proliferation medium consisting of DMEM (Thermo Fisher, #31,966,021) and Hams F12 (Thermo Fisher, #31,765,027) (2:1) supplemented with 2% B27 (Thermo Fisher, #17,504,044), 1% penicillin and streptomycin (Pan-Biotech, #P06-07,100), 20 ng/mL EGF (Thermo Fisher, #PHG0315) and either 20 ng/mL FGF (R&D Systems, #233-FB) for hNPCs or 10 ng/mL FGF (R&D Systems, #3339-FB-025) for rNPCs. Neurospheres were cultivated under standard culture conditions at 37 °C with 5% CO_2_ in cell culture dishes coated with poly(2-hydroxyethyl methacrylate) (poly-HEMA; Merck, #P3932). Once a week, neurospheres were passaged mechanically to 0.2-mm size using a McIlwain tissue chopper (model TC752), and thrice a week, half of the medium was replaced.

### Assay conditions and chemical exposure

Neurospheres were chopped to 0.2 mm 3 days before plating to reach a defined size of 0.3 mm. Spheres were plated in five technical replicate wells/condition in 96-well plates (flat bottom, Greiner, # 655,180) with one sphere/well plated in 100 µL of differentiation medium containing 13 concentrations of CHMs: 0.000001, 0.00001, 0.0001, 0.001, 0.0025, 0.005, 0.01, 0.025, 0.05, 0.075, 0.10, 0.50, and 1.0 mg/mL LGT and 0.000001, 0.00001, 0.0001, 0.001, 0.0025, 0.005, 0.01, 0.1, 0.25, 0.50, 0.75, 1.0, and 2.0 mg/mL TM. The differentiation medium composition was DMEM (Thermo Fisher, #31,966,021), Hams F12 (Thermo Fisher, #31,765,027) 2:1 supplemented with 1% of N2 (Thermo Fisher, #17,502–048) and 1% penicillin and streptomycin (Pan-Biotech, #P06-07,100). Neurospheres were exposed for 3 (for migration/adhesion endpoints) or 5 (for differentiation endpoints) days and all experiments were performed under standard culture conditions at 37 °C with 5% CO_2_. For the latter, on day 3, half of the exposure/solvent medium was exchanged and the supernatant was used to detect cytotoxicity by measuring lactate dehydrogenase (LDH) leakage.

### Migration, adhesion, and differentiation of NPCs

Upon removal of growth factors and plating neurospheres onto a poly-d-lysine (PDL, 0.1 mg/mL, Merck, #P0899) and laminin (0.0125 mg/mL, Merck, #L2020)-coated 96-well plate in differentiation medium, spheres settle down and NPCs migrate radially out of the sphere core. During migration NPCs simultaneously differentiate into radial glia, neurons, astrocytes, and oligodendrocytes.

#### Migration and adhesion

Radial glia migration distance was assessed after 72 h by taking brightfield images and manual measurement of the distance between the sphere core and the furthest migrated cells as number of pixels which is converted to µm. The endpoint-specific control for NPC2 was the src kinase inhibitor PP2 (Moors et al. 2007; Baumann et al. 2015) significantly reducing migration (data not shown). As cell migration requires cell adhesion and motility, time-lapse microscopy using a widefield system (EVOS FL Auto2, Thermo Fisher Scientific) was performed to record hNPC movement. For the time-lapse experiment, a 10 × air objective was used and images were recorded every 5 min over a time course of 24 h. Finally, videos were produced with the integrated software FL Auto2.

#### Immunocytochemical stainings

After 3 (for migration/adhesion endpoints) or 5 (for differentiation endpoints) days of migration, NPCs were fixed with a final concentration of 4% paraformaldehyde (PFA; Merck). The 96-well plates were incubated for 30 min at 37 °C and directly afterwards washed three times for 3 min with 250 µL PBS (-/-; Biochrom) and stored in PBS at 4 °C until immunostaining was performed. Cells had to be always covered with at least 40 µL PBS to prevent cell detachment. A blocking solution (PBS, 10% Goat Serum (GS; Sigma-Aldrich)), 10 µl/well was added and incubated for 15 min at 37 °C. After removal of blocking solution, cells were stained as follows:

Neurospheres (migrated for 3 days) were incubated overnight at 4 °C with a rabbit IgG anti-GFAP antibody solution (1:100, Sigma-Aldrich, #G9269; in PBS-T (PBS containing 0.1% Triton X-100) and 10% GS), followed by three 3-min washing steps by addition and removal of 250 µL PBS. After removal of PBS, a secondary antibody solution in PBS (1:200 Alexa Fluor 546 anti-rabbit IgG (Invitrogen, #A11010), 2% GS and 1% Hoechst 33,258 (Sigma-Aldrich, #B1155)) was added for 30 min at 37 °C. After washing steps as previously described, plates were stored in the dark at 4 °C until further analysis.

Neurospheres (migrated for 5 days) were incubated overnight at 4 °C with a mouse IgM oligodendrocyte O4 antibody solution (1:400 in PBS with 10% GS; R&D System, #MAB1326). After 1^st^ antibody incubation, cells were washed three times with 250 µL PBS for 3 min and a secondary antibody solution in PBS (1:400 Alexa Fluor 488 anti-mouse IgM (Thermo Fisher, #A-21042) and 2% GS was added for 30 min at 37 °C followed by washing steps and a second fixation with 4% PFA for 30 min at 37 °C. After three additional washing steps, cells were permeabilized in 0.1% PBS-T for 5 min at room temperature, followed by a blocking step for 15 min at 37 °C with PBS and 10% Rabbit Serum (RS; Thermo Fisher, #10,510). For neuronal co-staining, neurospheres were incubated for 1 h at 37 °C with a conjugated rabbit TUBB3 674 antibody (Abcam, #190,575) 1:400 (in PBS with 2% RS and 1% Hoechst 33,258). After washing steps, 250 µL PBS was added to each well and the plates were stored in the dark at 4 °C.

Imaging of immunochemical stainings was performed by high-content imaging analysis (HCA) using an automated fluorescence microscope (Cellomics ArrayScan VTI, Thermo Fisher Scientific). Respective channels (386 nm for Hoechst-stained nuclei, 546 nm for GFAP-stained radial glia cells and astrocytes, 647 nm for β(III)-tubulin-stained neurons, 488 nm for O4-stained oligodendrocytes) were acquired with a 200-fold magnification and a resolution of 552 × 552 pixel. The image analysis was performed with the HCA tool Omnisphero (Schmuck et al. 2017).

#### Differentiation

Differentiation into neurons and oligodendrocytes was determined as the number of all β(III)-tubulin- and O4-positive cells in percent of the total count of Hoechst-positive nuclei. Therefore, for two defined areas (1098 mm × 823 mm size; placed on opposite sides of the sphere core)/migration area, the numbers of β(III)-tubulin- and O4-positive cells were counted manually and then normalized to the total number of nuclei. Nuclei were counted automatically by using HCA (Cellomics ArrayScan VTI, Thermo Fisher Scientific). Due to specific hNPC migration endophenotype triggered by LGT, HCA could not annotate single nuclei at the concentration of 0.025 mg/mL LGT. Here, nuclei were counted manually by expert judgment. Therefore, all migrated and Hoechst-positive nuclei were counted and annotated by using ImageJ. The resulting percentages of β(III)-tubulin- and O4-positive cells within the two areas from the same sphere were pooled and the mean was calculated for the five neurospheres of each treatment. The growth factor EGF and bone morphogenetic protein 7 (BMP7) were used as endpoint-specific controls (Baumann et al. 2015) as they significantly reduced the total number of neurons and oligodendrocytes, respectively (data not shown).

### Viability and cytotoxicity

Viability and cytotoxicity assays were multiplexed within the experiment to distinguish specific compound effects from secondary effects due to loss of mitochondrial reductase activity and cytotoxicity. Mitochondrial reductase activity was assessed by an alamar blue assay (CellTiter-Blue assay (CTB); Promega) in the last 2 h of the compound treatment period. Cytotoxicity of treated NPCs was detected by measuring LDH (CytoTox-ONE membrane integrity assay; Promega) after 3 and 5 days of migration/differentiation. Both assays were performed according to the manufacturer’s instructions and as published previously (Nimtz et al. 2019). The relative fluorescence unit (RFU) values of the replicates were averaged and medium without cells was used to correct for background fluorescence. A reduction in radial glia migration decreases the CTB signal due to a diminished cell number in the migration area without necessarily affecting cell viability (Fritsche et al. 2018a). Thus, it is to note, when radial glia migration is inhibited by a compound, the LDH assay is the sole cell death reference assay for DNT specificity.

### Microarray analysis

For microarrays analysis, 1000 neurospheres with a defined size of 0.1 mm were plated per well of a PDL/laminin-coated 6-well plate and treated for 6 and 24 h with LGT and EGCG, and 60 h with TM. These time points were chosen based on the endpoints of concern affected within the “Neurosphere Assay” under respective compound exposure. hNPC migration directly starts after plating the neurospheres onto the extracellular matrix and within the first 24 h hNPC migration dynamic is the highest (Baumann et al. 2015; Koch et al. 2022). The same refers to the time point chosen for the TM-related oligodendrocyte effect. Sixty hours post plating, oligodendrocyte-related gene expression (*PLP1 and MBP*) increases 20–30-fold despite the low numbers of oligodendrocytes at this time point (Koch et al. 2022). Hence, this dynamic time point is suitable for observing adverse effects on oligodendrocyte development (Klose et al. 2021a).

The RNA isolation was performed using the RNeasy Mini Kit (Qiagen, #74,106) according to the manufacturer’s protocol. Afterwards, the total RNA was quantified (Qubit RNA HS assay, Thermo Fisher Scientific) and the quality was measured by capillary electrophoresis on a fragment analyzer using the “Total RNA Standard Sensitivity Assay” (Agilent Technologies, Inc., Santa Clara, USA). All samples had high RNA quality numbers (RQN; mean = 9.9).

cDNA synthesis, complementary RNA (cRNA conversion) synthesis, and subsequent biotin labeling of cRNA were performed according to the manufacturer’s protocol (GeneChip® WT PLUS Reagent Kit 703,174 23. January 2017; Thermo Fisher). Briefly, 100 ng of total RNA was converted to cDNA. After in vitro transcription into cRNA and 2^nd^ cycle synthesis, cDNA was fragmented and biotin labelled. Finally, end-labelled cDNA was hybridized to Applied Biosystems™ Clariom™ S Human Gene Expression Microarray chips for 16 h at 45 °C, stained with a streptavidin/phycoerythrin conjugate, and scanned as described in the manufacturer’s protocol.

For validation of microarray experiments, quantitative real-time polymerase chain reactions (qRT-PCR) of a set of 10 genes were performed (Suppl. Fig. S2) with the QuantiFast SYBR Green PCR Kit (Qiagen, # 204,054) using a Rotor-Gene Q Cycler (Qiagen). Therefore, 500 ng RNA from microarray samples was transcribed into cDNA using the RNeasy Mini Kit (Qiagen, #74,106) and the Quantitect Reverse Transcription Kit (Qiagen, #205,313) according to the manufacturer’s instructions. Analysis was performed using the software Rotor-Gene Q Series version 2.3.4 (Qiagen). Accordingly, copy numbers (CN) of the genes of interest were calculated by using gene-specific copy number standards as described previously in detail (Walter et al. 2019) and normalized to the housekeeping gene *BETA-ACTIN*. Selected genes and respective primer sequences are given in supplementary Table S3.

### DCFDA ROS assay

Accumulation of reactive oxygen species (ROS) was measured using 2′,7′-dichlorofluorescein diacetate (DCFDA; Sigma-Aldrich, #D6883). Therefore, 35 neurospheres with a defined size of 0.1 mm were plated per well of black/clear bottom PDL/laminin-coated 96-well plates (Thermo Fisher, #165,305) and treated with TM for 60 h. As a positive control, cells were incubated with 0.01 mM H_2_O_2_ for 45 min at 37 °C and 5% CO_2_. After treatments, hNPCs were washed with 100 µL prewarmed PBS and cultured in 100 µL differentiation medium containing 50 µM DCFDA for 30 min at 37 °C and 5% CO_2_. Afterwards, hNPCs were washed on ice using 100 µL refrigerated PBS and fluorescence was determined at 493em/522ex on a Tecan Infinite M200 Pro reader.

### Proliferation

A detailed description of the methods used to evaluate proliferation is given in the supplementary Material and Method section.

### Data analysis and statistics

All results are presented as mean ± standard error of the mean (SEM) from a minimum of at least three independent biological replicates. Independence is defined as experiments performed with NPCs from different individuals or from a different passage of cells. In case of 0.0025, 0.005, 0.025, 0.050, 0.075, and 0.5 mg/mL LGT and 0.0025, 0.005, 0.25, 0.50, 0.75, and 2 mg/mL TM, only one individual could be used and here independence is resulted from different passages. For concentration–response curves, a sigmoidal (variable slope) curve fit was applied using GraphPad Prism 8.2.1. Statistical significance was calculated with one-way ANOVA or two-way ANOVA followed by Bonferroni’s post hoc tests using the same software and results with *p*-values ≤ 0.05 were termed significant. Respective information is given in each figure legend.

Data analyses of Microarray CEL files was conducted with GeneSpring GX software (Vers. 14.9.1; Agilent Technologies). Probes within each probe set were pooled by the GeneSprings’ ExonRMA16 algorithm after quantile normalization of probe-level signal intensities across all samples, leading to a reduction of inter-array variability (Bolstad et al. 2003). The process of data input was concluded by baseline transformation to the median of all samples. After grouping of samples (4 technical replicates each) according to their respective experimental condition, a given probe set had to be expressed above background (i.e., fluorescence signal of that probe set was detected within the 20^th^ and 100^th^ percentiles of the raw signal distribution of a given array) in all 4 replicates in at least one of the conditions to be further analyzed in pairwise or ANOVA comparisons. Statistical significance was calculated with moderate *t*-tests or one-way ANOVA (*p* ≤ 0.05 was termed significant) followed by Benjamini–Hochberg tests. The overrepresented gene ontology (GO) enrichment analysis was performed using the online tool DAVID Bioinformatics Resources 6.8 (DAVID). Therefore, genes with *p* ≤ 0.05 and fold change ≥ 1.5 (LGT and EGCG), ≥ 2 (TM) were termed differentially expressed (DEX).

## Results and discussion

In the last decades, the consumption of CHMs during pregnancy has been increasing not only in Asian countries, but also worldwide. There are over 60% and 45% pregnant women in Canada (Hollyer et al. 2002) and in the USA (Glover et al. 2003), respectively, using CHMs during their pregnancy, while the consumption in European countries averages to up to 20% (Hemminki et al. 1991; Glover et al. 2003; Nordeng and Havnen 2004). However, the hazard of most CHMs has not been thoroughly investigated, since safety evaluations are commonly performed in in vivo studies (Li et al. 2019b). Especially DNT in vivo hazard assessments are extremely resource-intensive; require high amounts of animals, time, and money; and are thus insufficient for large-scale testing (Lein et al. 2005; Crofton et al. 2012). This is one of the reasons why they are not mandatory for safety assessment of compounds in general, including CHMs. In addition, there are some uncertainties in their methodology, evaluation, and regulation and they bear the issue of species extrapolation (Tsuji and Crofton 2012; Terron and Bennekou Hougaard 2018; Sachana et al. 2019). To cover the need for DNT testing for regulatory purposes, the OECD is currently supporting the delivery of a guidance document (Crofton and Mundy 2021) that will facilitate the use of DNT-NAMs within a DNT-IVB in an IATA context (Sachana et al. 2021). This guidance document is supported by case studies including mechanistic evaluation of different compound classes in the DNT-IVB in a regulatory context (Sachana et al. 2021). The present study extends the OECD case studies by investigating the DNT effects of two CHMs, which belong to a totally different compound class, in test methods of the current DNT-IVB setup to assess the DNT effects of two CHMs on the neurodevelopmental KE NPC proliferation, NPC migration and adhesion, and differentiation into neurons and oligodendrocytes in a 3D neurosphere-based in vitro model consisting of human and rat NPCs (Baumann et al. 2014; Barenys et al. 2017a; Masjosthusmann et al. 2018, 2020; Nimtz et al. 2019; Klose et al. 2021a; Sachana et al. 2021).

LGT is classified as a toxic herb in the Chinese Pharmacopoeia (Chinese Pharmacopoeia Commission 2020), as it is known to be toxic to humans, rats, dogs, pigs, and insects, but non-toxic to sheep, rabbits, cats, and fish (Chinese Pharmacopoeia Commission 2020; Zhang et al. 1984). Toxicity was reported for the gastrointestinal system (Liu et al. 2018a), the central nervous system (hypothalamus, midbrain, medulla, cerebellum, and spinal cord; Wang et al. 2016), and the cardiovascular system (Wang et al. 2020), while bleeding and necrosis of the liver were also observed (Li et al. 2020b). LGT is mainly used as a treatment for leprosy, rheumatoid arthritis, tuberculosis, and other chronic lung disorders (Chen 2001; Wang et al. 2018; Song et al. 2020a). Contrary, TM is classified as a non-toxic herb, as in vivo *s*tudies showed its safety in acute and sub-acute toxicity tests (Yuan et al. 2013; Zhan et al. 2016), with no records about its safety in pregnancy so far. It has a wide clinical application including headaches, dizziness, neurasthenia, facial cramps, limb numbness, hemiplegia, epilepsy, hypertension, and tetanus (Dai et al. 2017; Liu et al. 2018b; Chen et al. 2019). Furthermore, TM is associated with insomnia relief, heart protection, memory enhancement, neuronal system protection, and improvement of synaptic plasticity (Manavalan et al. 2012; Wang et al. 2014; Zhan et al. 2016; Lin et al. 2017; Huang et al. 2018). Therefore, in the present study, we used LGT as a potentially hazardous CHM, while the neurodevelopmental consequences of TM had not yet been studied. For both compounds, their DNT-potential and especially their MoAs had not been characterized so far. Due to their broad indications, their consumption during pregnancy cannot be excluded. A detailed list of LD_50_ values for both compounds can be found in the supplementary material (Suppl. Table S1). Both TM and LGT are commercially available and usually dissolved in a cup of hot water, hence orally consumed.

### *LGT disturbs fundamental KEs of human NPC development *in vitro

Proliferation of NPCs determines brain size (De Groot et al. 2005), illustrating an essential neurodevelopmental KE. In order to assess the impact of LGT on hNPC proliferation, we found that 0.1 mg/mL LGT significantly reduced hNPC proliferation measured by sphere size increase as well as BrdU incorporation into the DNA to 65.8% ± 5.2% and 30.4% ± 2.9% of untreated controls, respectively, without reducing viability or triggering cytotoxicity (Suppl. Fig. S1). The highest concentration of LGT tested (1 mg/mL) leads to a total inhibition of both hNPC proliferation measurements. However, also viability and cytotoxicity were significantly affected at this concentration (Suppl. Fig. S1).

**Fig. 1:**
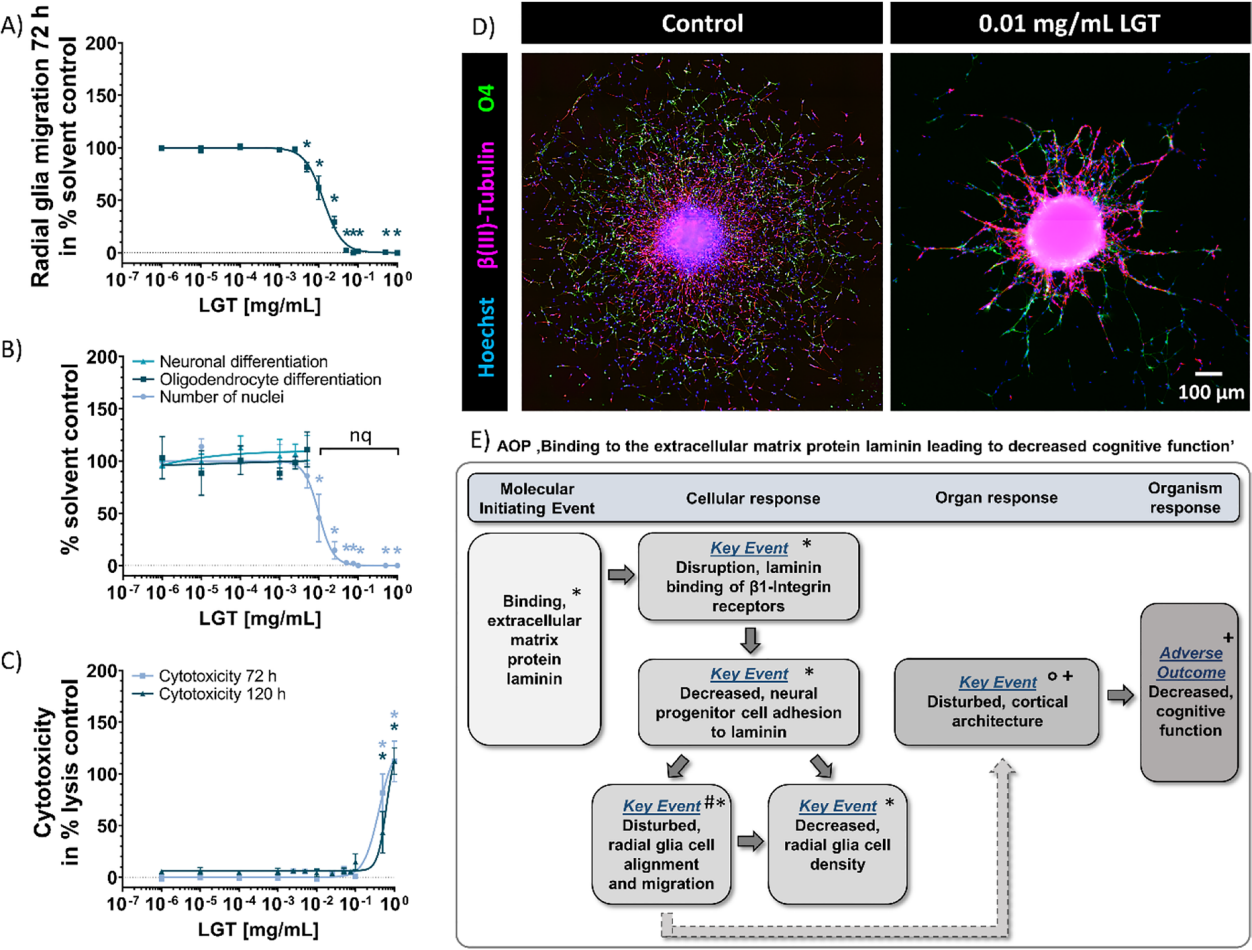
Effects of LGT on hNPC migration and differentiation supporting the AOP “Binding to the extracellular matrix protein laminin leading to decreased cognitive function.” Spheres with a defined size of 0.3 mm were plated for hNPC migration analyses onto poly-d-lysine/laminin-coated 96-well plates in presence and absence of LGT for 120 h. Radial glia migration (72 h) was determined by manually measuring the radial migration from the sphere core (**A**). Differentiation into neurons and oligodendrocytes was determined by performing immunocytochemical stainings and using the software Omnisphero (Schmuck et al. 2017). The number of all β(III)-tubulin-positive (red) and O4-positive (green) cells in the migration area after 120 h of differentiation were counted manually and their percent of Hoechst-positive nuclei (blue) was calculated (**B**, **D**). In parallel, cytotoxicity (**C**) was assessed by the LDH assay. At least three independent experiments with 5 technical replicates were performed and presented as mean ± SEM. Statistical significance was calculated using one-way ANOVA followed by Bonferroni’s post hoc tests, *significant compared to the solvent control (*p* ≤ 0.05 was considered significant). (**E**) The schematic AOP “Binding to the extracellular matrix protein laminin leading to decreased cognitive function” includes the laminin-dependent decreased adhesion of NPCs as a central key event resulting in cortical architecture alterations and adverse outcomes in the developing brain. This AOP is based on published results: *Barenys et al. 2017b, a and Kühne et al. 2019; ^#^Graus-Porta et al. 2001; °Belvindrah et al. 2007; ^+^Amin and Borrell 2020, Fernández et al. 2016, and Long and Huttner 2019. nq, not quantifiable (relevant for number of neurons and oligodendrocytes)

Another fundamental neurodevelopmental KE is NPC migration. Cortex development takes place during the fetal phase of brain development and involves radial glia migration leading to the formation of a scaffold that is especially used by neurons to migrate and build the cortical layers ensuring normal brain structure and function (Borrell and Götz 2014). LGT exposure disturbed hNPC migration as the most sensitive endpoint (MSE; Fig. 1A, D) across the battery of test methods, since LGT significantly reduced radial glia migration distance (72 h) at concentrations between 0.005 mg/mL and 0.1 mg/mL to 81.6 ± 4.4% and 1.5 ± 0.2% of controls (Fig. 1A), without inducing cytotoxicity (Fig. 1C). While 0.5 mg/mL inhibited hNPC migration almost completely to 0.4 ± 0.2% of controls, the highest tested concentration of 1 mg/mL LGT leads to a total inhibition of migrated radial glia cells. However, also cytotoxicity was significantly induced at these concentrations to 81.7 ± 18.1% and 112.0 ± 19.7% (72 h) as well as to 43.4 ± 20.0% and 112.3 ± 12.7% (120 h) of lysis controls, respectively (Fig. 1C). Similar to the migration distance measurements shown in Fig. 1A, LGT significantly reduced the number of migrated Hoechst-positive nuclei after 120 h in a concentration-dependent manner (Fig. 1B). Here, 0.01 mg/mL LGT significantly inhibited nuclei numbers to 45.5 ± 22.8% of untreated controls, while ≥ 0.1 mg/mL LGT caused a complete loss of nuclei due to absence of migration. The decreased number of nuclei serves as a second readout for the disturbed cell migration. Within the migration area, hNPCs differentiate into different effector cells of the human brain, e.g., neurons, astrocytes, and oligodendrocytes (Schmuck et al. 2017). To analyze the influence of LGT on hNPC neuronal and oligodendrocyte differentiation, all β(III)-tubulin- and O4-positive cells in percent of Hoechst-positive nuclei in the migration area after 120 h of differentiation were quantified (Fig. 1B). Under influence of LGT (≤ 0.005 mg/mL), differentiation into neurons and oligodendrocytes was not affected. Neurons and oligodendrocytes were not quantifiable after exposure to ≥ 0.01 mg/mL LGT due to the altered phenotype of the migration area (Fig. 1D) displaying an irregular migration pattern containing gaps and arborized structures with cells seeming to adhere to each other.

Cell migration requires cell adhesion as well as cell motility. In order to investigate whether LGT affects hNPC movement or adhesion, we next performed time-lapse microscopy of plated neurospheres over a time course of 24 h (Online Resource). The videos clearly demonstrate that 0.01 and 0.1 mg/mL LGT did not affect hNPC motility but their adhesion, since cells try to attach to the extracellular matrix (ECM) and upon unsuccessful attachment migrate back into the sphere. This impaired adhesion causes an irregular migration pattern with gaps and arborizations similar to Fig. 1D.

This specific neurosphere “gap and arborization” endophenotype was previously observed upon neurosphere treatment with the flavonoid EGCG (Barenys et al. 2017a), the most abundant catechin in green tea (Rothwell et al. 2013).

Based on these data in human and rat NPCs (Barenys et al. 2017a), we generated the putative AOP “Binding to the extracellular matrix protein laminin leading to decreased cognitive function” (Bal-Price et al. 2016; Fig. 1E), which was submitted to the OECD in 2019. The MIE identified in this AOP is the interference of a compound with the ECM protein laminin, thereby disturbing binding of NPCs via their β1-integrin receptors to laminin. Laminin is a major component of the brain’s extracellular matrix and is essential for normal brain development and function (Chen et al. 2009). The in vivo relevance of β1-integrin function for cortical development was demonstrated in conditional β1-integrin-deficient (CNS-(nestin-Cre)-β1-integrin-deficient) mice. These animals display defects in the organized laminar cytoarchitecture of cortical structures due to defective anchoring of glial endfeet (Graus-Porta et al. 2001). In vitro, nestin-Cre-β1-integrin-deficient glia cells from these mice do not develop cell processes with their typical radial glia fibers (Belvindrah et al. 2007), suggesting that the transmembrane β1-integrin complex regulates glial process outgrowth and endfeet anchorage, a prerequisite for neuronal migration and positioning. Lately, the significant modulating effect of the ECM including laminin and integrins for cortex morphogenesis, especially for gyrencephalic species like humans, has been appreciated (Long and Huttner 2019; Amin and Borrell 2020). Hence, the AO of this AOP is “Decreased cognitive function” as a result of disturbed cortical radial glia alignment and migration, which are pathognomonic for disturbed cortical architecture with impaired cortical folding during development that leads to severe intellectual disability when caused by gene mutations (Fernández et al. 2016). Induction of this phenotype was recently used for prioritization of EGCG analogues for possible clinical application (Kühne et al. 2019).

### *LGT disturbs radial glia adhesion of human NPCs *in vitro* by interacting with the extracellular matrix glycoprotein laminin*

**Fig. 2:**
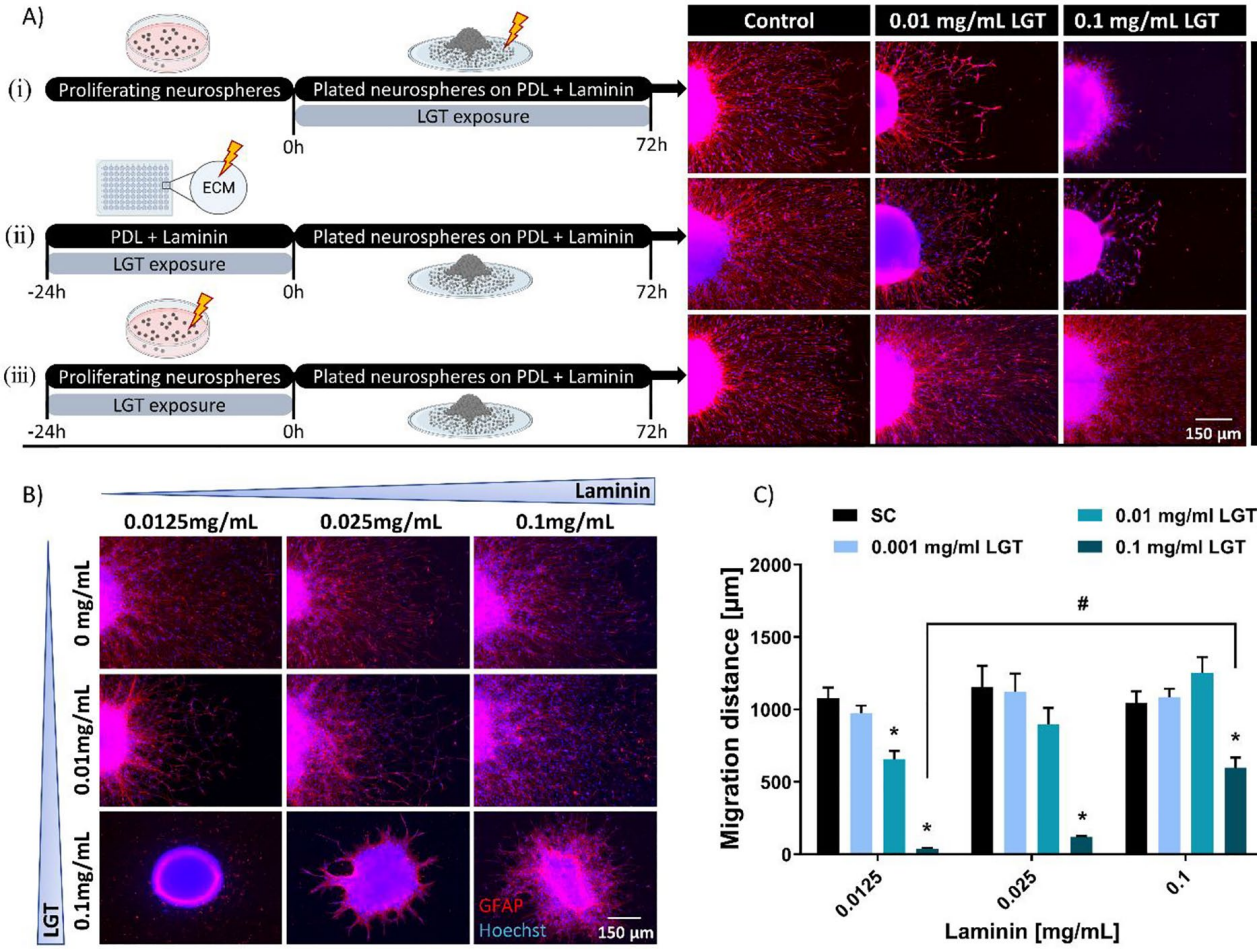
LGT disturbs migration by interacting with laminin. Spheres with a defined size of 0.3 mm were plated onto an extracellular matrix (ECM) consisting of poly-d-lysine (PDL) and laminin, and cultured for 72 h in presence and absence of LGT under following conditions: Spheres and ECM were exposed to LGT for 72 h during the whole migration period; ECM was pre-exposed to LGT for 24 h and used to culture spheres without LGT; spheres were pre-exposed to LGT for 24 h and cultured without LGT on a non-treated ECM (**A**). To visualize the migration area and radial glia orientation, immunocytochemical stainings of GFAP-positive cells (red) and Hoechst-positive nuclei (blue) were performed. Representative immunocytochemical stainings (**B**) and measurement of the migration distance in µm (**C**) of hNPC exposure to increasing LGT and laminin concentrations. Data shown in (C) are derived from at least three independent experiments with 5 technical replicates and presented as mean ± SEM. Statistical significance was calculated using one-way ANOVA followed by Bonferroni’s post hoc tests (*p ≤ 0.05 was considered significant) and two-way ANOVA followed by Tukey’s multiple comparison test (^#^*p* ≤ 0.05 was considered significant). *Significant compared to respective solvent control; ^#^significant compared to the 0.1 mg/mL LGT treatment plated on 0.0125 mg/mL laminin. Schematic experimental setup (**A**) was created with BioRender.com

To elucidate if also LGT produces—similar to EGCG (Barenys et al. 2017a)—the migration endophenotype of “gap and arborization” by binding to laminin and thereby disrupting laminin-integrin binding, we performed experiments with three different LGT exposure scenarios: (i) spheres and ECM were exposed to LGT during the whole migration period of 72 h, (ii) the ECM was pre-exposed to LGT for 24 h prior to plating LGT-unexposed spheres, and (iii) proliferating spheres were pre-exposed to LGT for 24 h and plated in absence of LGT on an unexposed laminin matrix (Fig. 2A).

For visualization of the migration pattern as well as the radial glia orientation, we performed immunocytochemical stainings of GFAP^+^ cells. The alterations in the migration phenotype are only observed when the ECM was exposed or pre-exposed to LGT (Fig. 2A, conditions i + ii) and not when only the spheres and not the ECM were pre-exposed to the compound and plated onto an unexposed ECM (Fig. 2A, condition iii). These results suggest that LGT binds to laminin, thereby prohibiting laminin-cell surface receptor interaction, leading to a disturbed cell adhesion. To confirm this hypothesis, we performed LGT/laminin co-exposure experiments (Fig. 2B, C). Increasing the assay’s laminin concentration in the “Neurosphere Assay” from 0.0125 mg/mL to 0.025 mg/mL and 0.1 mg/mL antagonized the LGT-induced migratory endophenotype (Fig. 2B) and the decreased migration distance (Fig. 2C) in a concentration-dependent manner. 0.1 mg/mL laminin rescued the strong, LGT-induced reduced migration distance (39.4 ± 5.0 µm) to 597.0 ± 71.4 µm, yet did not completely compensate the adverse effect, while 0.025 mg/ml laminin completely antagonized the migration inhibition caused by 0.01 mg/ml LGT.

Cell adhesion to the ECM protein laminin primarily depends on integrins, which are composed of a family of α/β heterodimeric transmembrane receptors which are responsible for the cell adherence to the ECM and take part in specialized cell–cell interactions (Hynes 2002). Integrin dimers containing β1- and β4-subunits are known to be responsible for laminin binding, with the β1-subunit playing the major part (Humphries et al. 2006; Barczyk et al. 2010). Chemicals that have a binding affinity to the ECM protein laminin mask the laminin-β1 and/or β4-integrin receptor and are therefore suggested to affect important cellular key aspects of neurodevelopment including cell adhesion, cell orientation, and cell migration thus disturbing cortical development (Graus-Porta et al. 2001; Belvindrah et al. 2007; Tzu and Marinkovich 2008; Warren et al. 2012; Lubbers et al. 2014; Barenys et al. 2017a).

That hNPC adhesion is also mainly dependent on β1-containing receptors was shown earlier by Barenys et al. (2017a). In this study, RT-PCR analyses revealed mRNA expression of β1- and β4-integrin subunits in migrating hNPCs. By using functional blocking antibodies against β1-integrin, we observed a decrease in hNPC migration and an irregular migration pattern, while a functional blockage of β4-integrin did not result in altered hNPC migration (Barenys et al. 2017a). This migration endophenotype of “gaps and arborization” seems to be specific for disturbed hNPC adhesion, because compounds inhibiting migration due to alterations of intracellular signaling like methylmercury chloride (MeHgCl) or the src kinase inhibitor PP2 decrease hNPC migration without causing gap formation in the migration area (Moors et al. 2007; Fritsche et al. 2018a; Masjosthusmann et al. 2018). Due to the striking similarity with the endophenotype observed after EGCG treatment, we suggest that also the LGT-induced migration phenotype is caused by interference of the compound with binding of the β1-integrin subunit to laminin. The relevance of the previously described AOP (Bal-Price et al. 2016; Fig. 1E) for the LGT MoA is supported by experimental data, i.e., antagonization of LGT effects by laminin (Fig. 2C) and observation of the chaotic orientation of GFAP^+^ radial glia cells (Fig. 2A, B).

### Transcriptome analyses of migrating hNPCs exposed to LGT and EGCG

**Fig. 3:**
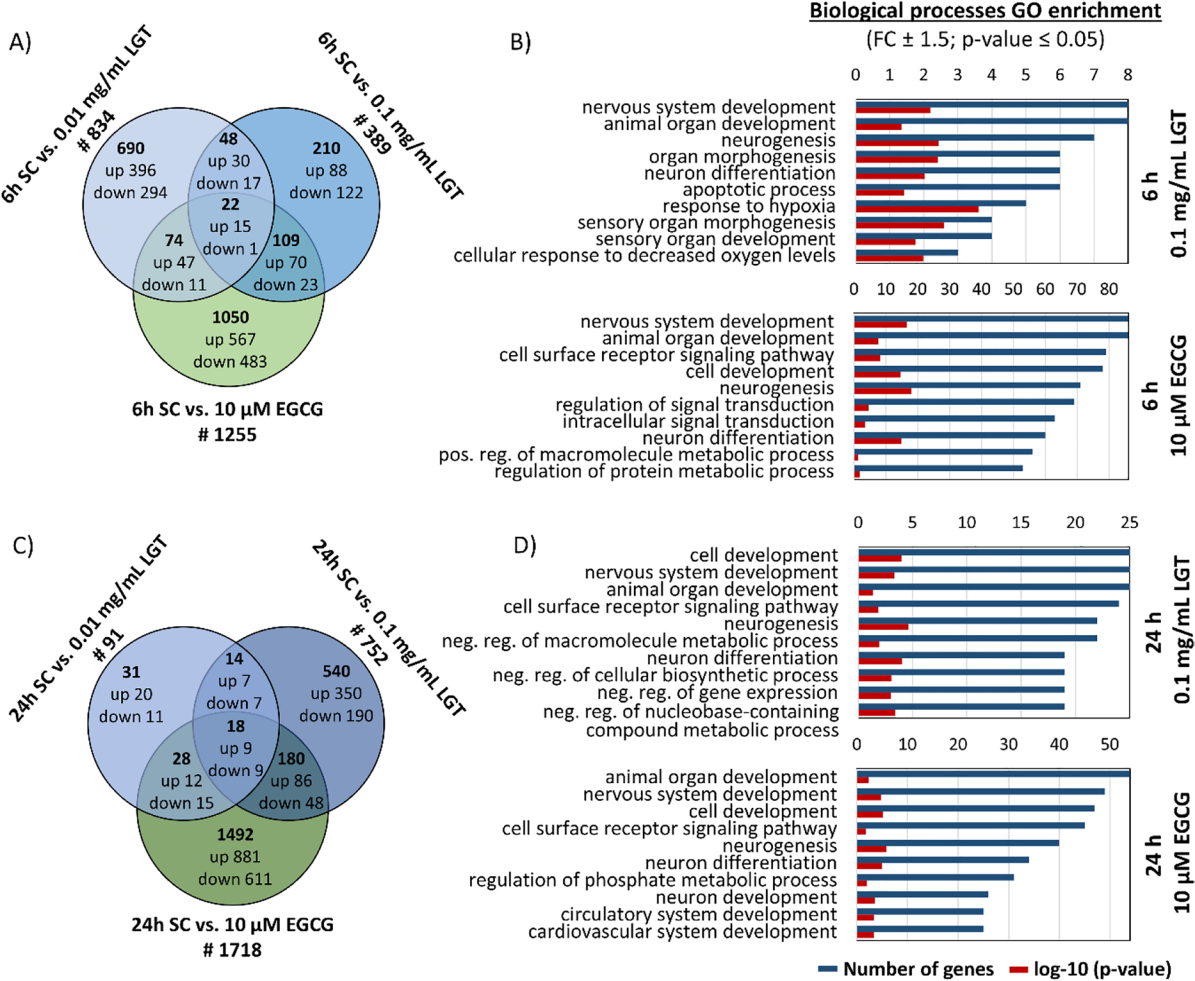
Transcriptome profiling of migrated and differentiated hNPCs treated with LGT and EGCG. Differential gene expression between hNPCs exposed to solvent (N2 Medium for LGT; DMSO for EGCG), LGT (0.01, 0.1 mg/mL) and EGCG (10 µM) over the time course (6 and 24 h) of differentiation was statistically determined using one-way ANOVA followed by Benjamini–Hochberg tests. Genes with *p* ≤ 0.05 and fold change ≥ 1.5 were termed differentially expressed (DEX). Overlap of the number of DEX genes regulated by LGT and EGCG over the time course of 6 h (**A**; SC vs. 0.01 mg/mL LGT, #834; SC vs. 0.1 mg/mL LGT, #389; SC vs. 10 µM EGCG, #1255) and 24 h (**C**; SC vs. 0.01 mg/mL LGT, #91; SC vs. 0.1 mg/mL LGT, #752; SC vs. 10 µM EGCG, #1718) of hNPC differentiation. Overrepresented gene ontology (GO) terms for 6 h (**B**) and 24 h (**D**) of differentiated hNPCs under influence of LGT and EGCG. GO enrichment analysis was performed using the online tool DAVID Bioinformatics Resources 6.8 (DAVID). All overrepresented GO terms were sorted by their number of genes involved (blue bars). Additionally, the *p*-value of each GO term is given (red bars). GO terms with highest number of genes involved are displayed. SC, solvent control

To further elucidate the mechanism(s) underlying the LGT- or EGCG-induced migratory phenotype, we performed microarray analyses of hNPCs differentiated for 6 h and 24 h in presence of 0.01 mg/mL and 0.1 mg/mL LGT or 10 µM EGCG and their respective solvents (N2 Medium for LGT; 0.1% DMSO for EGCG; Fig. 3).

The Venn diagrams illustrate the total number of gene changes (Fig. 3A, C). Both concentrations of LGT (0.01 mg/mL and 0.1 mg/mL) as well as 10 µM EGCG significantly (*p* ≤ 0.01, fold change ≥ 1.5) regulated the expression of 834, 389, and 1255 genes, respectively, already after 6 h (Fig. 3A), indicating that transcriptome changes take place within the first hours of migration. After 24 h of migration, the number of significantly regulated genes increased to 752 and 1718 in 0.1 mg/mL LGT- and EGCG-treated spheres, respectively, while 0.01 mg/mL LGT regulated with 91 genes less than after 6 h (Fig. 3C). In general, EGCG regulates more genes than LGT after both time points measured. Both compounds commonly regulated only 22 and 18 genes after 6 h and 24 h, respectively (Suppl. Tab. S4). Strikingly, just one gene (cell adhesion molecule-related/downregulated by Oncogenes; *CDON*) within the 22 genes regulated after 6 h is directly associated with cell migration and/or adhesion, while after 24 h, none of the overlap genes is directly related to migration and/or adhesion processes. CDON has been identified as a Sonic hedgehog (Shh) receptor with affiliations to the neural cell adhesion molecule (N-CAM) family (Tenzen et al. 2006; Zhang et al. 2006). Specifically, CDON plays an important role during neuronal differentiation as it binds to N-cadherin, a cell adhesion molecule, thereby inducing p28/MAPK signaling to direct cell differentiation (Lu and Krauss 2010). Contrary to CDON upregulation caused by LGT (0.01 mg/mL FC 1.8; 0.1 mg/mL FC 2.0) and EGCG (FC 5.6), knockdown of CDON in zebrafish neural crest cells (NCCs) resulted in aberrant migration, as NCCs are still able to migrate out of the neural tube but delay directly after the initiation of migration (Powell et al. 2015). Furthermore, this zebrafish study illustrates via live cell imaging a reduced directedness of migration, increased velocity, and mispositioned cell protrusions. In our case here, it can be speculated that CDON expression might be a compensatory mechanism for the aberrant migration produced by compound exposure. This has to be more thoroughly studied in the future.

In accordance with these observations, gene ontology (GO) enrichment analyses and the ten highest overrepresented biological processes sorted by their number of genes involved revealed no enriched migration and/or adhesion process after 6 h and 24 h 0.1 mg/mL LGT and 10 µM EGCG hNPC (Fig. 3B, D). However, three of the enriched processes are directly brain-related (“nervous system development,” “neurogenesis,” and “neuron differentiation”) and overrepresented in all conditions. In addition, the GO term “neuron development” is present after 24 h of hNPC treatment with 10 µM EGCG (Fig. 3D). With the exception of 6 h 0.1 mg/mL LGT, there is one process “cell surface receptor signaling pathway” overrepresented in all conditions, which needs further attention regarding our findings of altered cell adhesion to the ECM. Here, EGCG had a greater impact since 79 (6 h) and 45 (24 h) genes are involved, while LGT led to a dysregulation of 24 genes relevant for this GO enrichment. Precisely, only nine genes in total (Receptor-type tyrosine-protein phosphatase T, *PTPRT*; Semaphorin 3E, *SEMA3E;* Integrin beta-5, *ITGB5*; Cadherin-6, *CADH6*; Membrane-associated guanylate kinase, *MAGI2*; Adhesion G protein-coupled receptors (GPCRs) latrophilin 2 and 3, *ADGRL2*, *ADGRL3*; Contactin-1, *CNTN1*; *CDON*) are directly associated with cell adhesion. As one possible MoA for disturbance of LGT-induced adhesion defects, we analyzed gene expressions associated with responses to ROS (oxidative stress induced growth inhibitor 1, *OSGIN1*; heme oxygenase 1, *HMOX1*; NAD(P)H dehydrogenase quinone 1, *NQO1*; glutamate-cystein ligase modifier subunit, *GCLM*; malic enzyme 1; *ME1*)*.* However, no significantly changes compared to untreated cells can be observed (Suppl. Fig. S3). One important process necessary for cell migration is G protein-coupled receptor stimulation (Cotton and Claing 2009), illustrating another possible MoA of LGT. Recently, Ding et al. (2021) indicated that LGT might cause reproductive toxicity by interacting with eleven genes associated to G protein-coupled receptor signaling pathway. However, none of these genes mentioned in this study that are affected by LGT is differential expressed in LGT-exposed migrated/differentiated hNPCs.

This lack of transcriptome data contributing to the knowledge on the MoA of LGT and EGCG is not surprising, considering that the common MIE “binding of a chemical to the extracellular matrix protein laminin” up to the KE “decreased adhesion of NPC to laminin,” ultimately leading to the observed migratory endophenotype, are orchestrated without genomic involvement. Therefore, we interpret the observed minor transcriptomic changes as secondary effects and compensatory responses of the cell to the defective cell–matrix adhesion/interaction and subsequently migration.

### Clinical observations concerning LGT

Strikingly, general toxicity of LGT exhibits enormous species differences in vivo, as it is toxic to humans, rats, dogs, pigs, and insects, but non-toxic to sheep, rabbits, cats, and fish (Chinese Pharmacopoeia Commission 2020; Zhang et al. 1984). Species differences might be caused by species-specificities in toxicodynamics or toxicokinetics (Dragunow 2020). As we did not observe species differences for EGCG potency on adverse human and rat NPC migration (Barenys et al. 2017a), it is highly likely that the adversity on NPC adhesion/migration observed in this study is also the underlying mechanism of the LGT in vivo toxicity in rats. In traditional Chinese medicine (TCM) clinics, the recommended therapeutic intake of LGT is 6 g/day (Chinese Pharmacopoeia Commission 2020) by oral administration, as LGT is usually dissolved in a cup of hot water. Because kinetics and bioavailability of this herbal compound mixture are not known, internal exposure data cannot be estimated.

This is the first time that the specific impact of the whole LGT extract on cell adhesion was shown in human cells in vitro, as most studies deal with single components and/or specific target-related in vivo models trying to evaluate single MoA. In a previous work from Kong et al. (2013), triptolide, a diterpenoid triepoxide LGT component, was shown to adversely affect matrigel-induced cell adhesion in human fibroblast-like rheumatoid arthritis synoviocytes and human umbilical vein endothelial cells. If the LGT-induced altered adhesion of hNPCs to laminin is also caused by triptolide is not known, however, it can be assumed since triptolide represents one of the main active component of LGT (He et al. 2013). To get a deeper understanding of prenatal LGT toxicity, further studies are needed, especially to identify and quantify the adhesion-disturbing component(s) in the LGT extract mixture we used. Moreover, single-component experiments and a better understanding of the in vitro and in vivo pharmacokinetics of the substance(s) are warranted. The latter will allow moving from hazard characterization to risk assessment.

### TM exposure disrupts oligodendrogenesis by producing oxidative stress

**Fig. 4:**
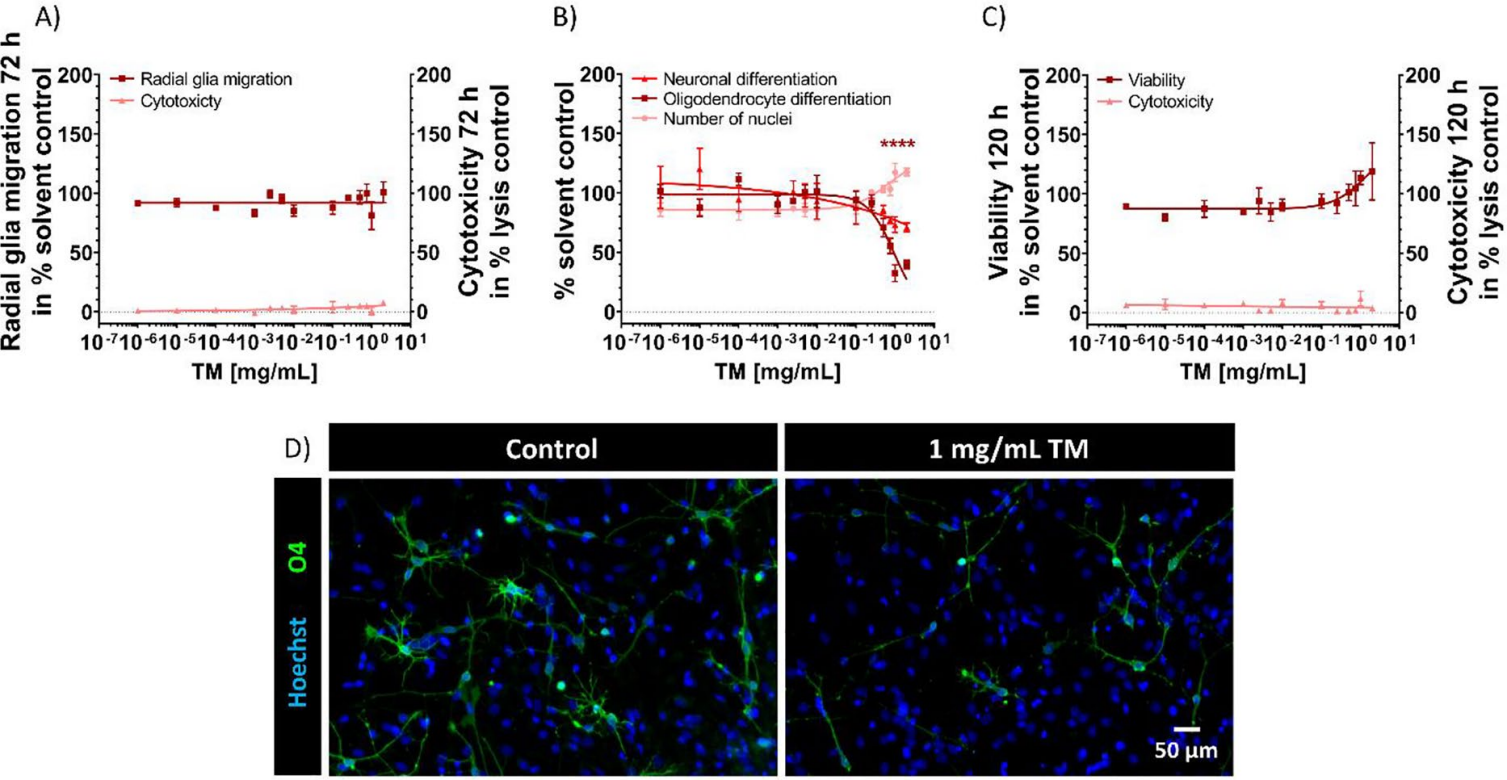
Effects of TM on hNPC migration and differentiation. Spheres with a defined size of 0.3 mm were plated onto poly-d-lysine/laminin-coated 96-well plates and exposed to increasing TM concentrations over 120 h. Radial glia migration (72 h) was determined by manually measuring the radial migration from the sphere core (**A**). Differentiation into neurons and oligodendrocytes was determined by performing immunocytochemical stainings and using the software Omnisphero (Schmuck et al. 2017). The number of all β(III)-tubulin-positive cells (**B**) and O4-positive cells (**B**, **D**; green) in percent of Hoechst-positive nuclei (blue) in the migration area after 120 h of differentiation was calculated manually. In parallel, viability and cytotoxicity (**A**, **C**) were assessed by the Alamar Blue and the LDH assay. At least three independent experiments with 5 technical replicates were performed and depicted as mean ± SEM. Statistical significance was calculated using one-way ANOVA followed by Bonferroni’s post hoc tests, *significant compared to the solvent control (*p* ≤ 0.05 was considered significant)

Next, we analyzed the impact of the second CHM, TM, on hNPC proliferation, radial glia migration, and differentiation into neurons as well as oligodendrocytes (Fig. 4). The proliferation of hNPCs, measured by the increase of sphere size and by BrdU incorporation into the DNA, was not significantly altered by TM concentrations up to 1 mg/mL over 72 h (Suppl. Fig. S1). Moreover, TM did not affect migration (Fig. 4A) or neuronal differentiation (Fig. 4B) at concentrations ≤ 2 mg/mL. However, ≥ 0.5 mg/mL TM significantly reduced the differentiation into oligodendrocytes to 70.7 ± 7.8% and 32.4 ± 7.0% (1 mg/mL TM) of controls (Fig. 4B, D) without significantly affecting the number of nuclei (Fig. 4B) or viability (Fig. 4C) or inducing cytotoxicity (Fig. 4A, C). In comparison to 1 mg/mL TM, doubled concentration (2 mg/mL TM) did not further decrease oligodendrocyte differentiation resulting in 39.7 ± 3.6% of untreated controls (Fig. 4B).

**Fig. 5:**
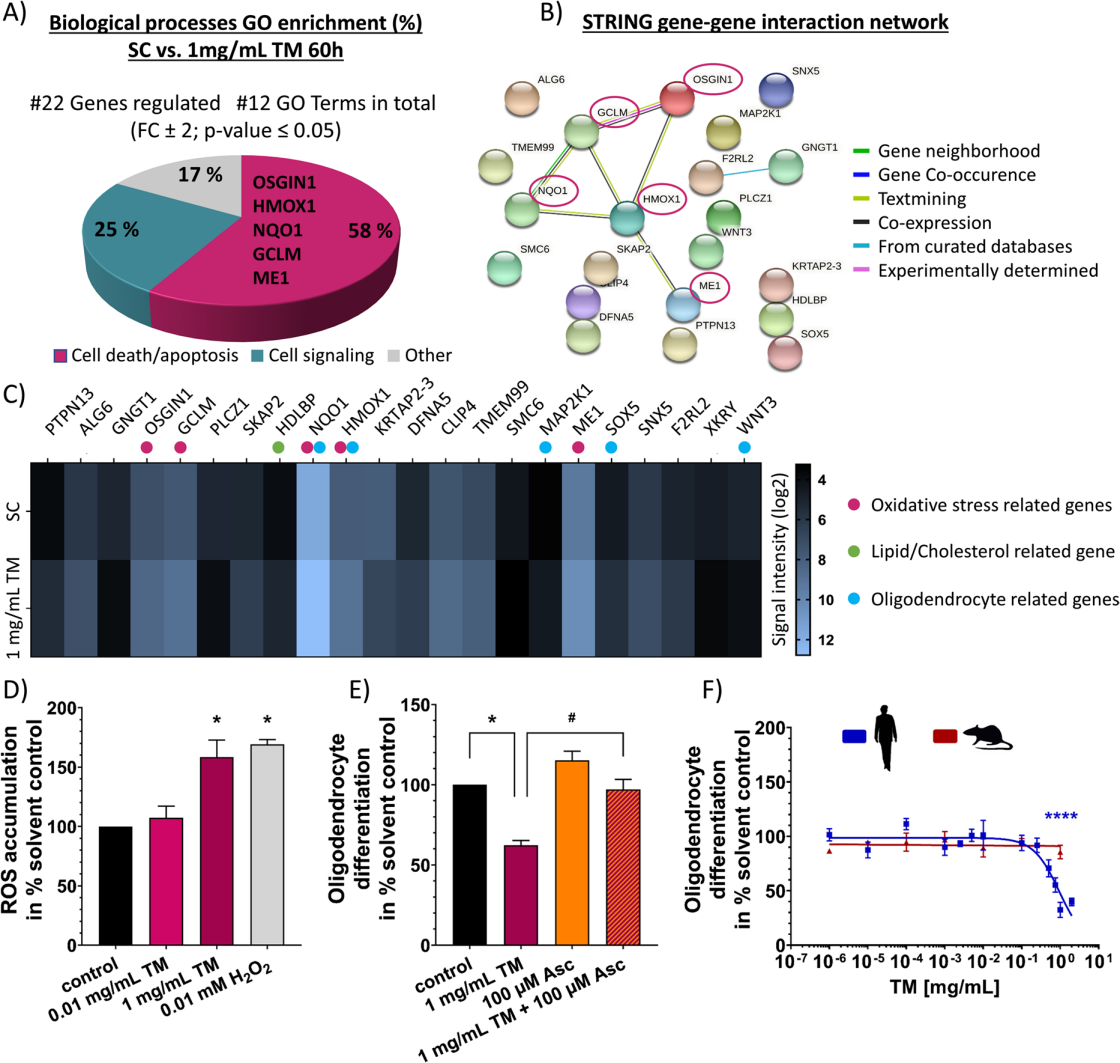
Transcriptomic profiling of differentiated hNPCs treated with TM. Differential gene expression between hNPCs exposed to 1 mg/mL TM and untreated hNPCs over 60 h of differentiation was statistically determined using moderated *t*-test followed by Benjamini–Hochberg tests. Genes (#22) with *p* ≤ 0.05 and fold change ≥ 2 were termed differentially expressed (DEX). Overrepresented gene ontology (GO) terms for hNPCs differentiated under the influence of 1 mg/mL TM for 60 h (**A**). GO enrichment analysis was performed using the online tool DAVID Bioinformatics Resources 6.8 (DAVID). The significantly regulated GO terms (#12) after 60 h of differentiation were further assigned to 3 superordinate processes based on expert judgment. Numbers in the pie chart represent the percentage of GO terms assigned to each superordinate process. Gene–gene interaction network analysis of the genes regulated by 1 mg/mL TM shows involvement of oxidative stress (**B**). Expression profile (absolute signal intensity) of the 22 differentially regulated genes identified in (**A**) between SC and 1 mg/mL (**C**). Genes are highlighted as reportedly regulated by oxidative stress (red dots), involved in lipid/cholesterol metabolism (green dot) or associated with oligodendrogenesis (blue dots). ROS accumulation (**D**) was measured via DCFDA oxidation over 60 h in hNPCs treated with 0.01 and 1 mg/mL TM. Exposure to 0.01 mM H_2_O_2_ was used as a positive control. hNPCs were exposed to 1 mg/mL TM, 100 µM ascorbic acid (Asc) alone and in combination (**E**). Human and rat spheres with a defined size of 0.3 mm were plated onto poly-d-lysine/laminin-coated 96-well plates and exposed to increasing TM concentrations over 120 h (**F**). The differentiation into oligodendrocytes (**E**, **F**) was determined as number of all O4-positive cells in percent of the total amount of Hoechst-positive nuclei in the migration area. At least three independent experiments with 5 technical replicates were performed and presented as mean ± SEM (in (**D**) TM treatment *n* = 4; H_2_O_2_ *n* = 3; in (**E**) control, TM and co-treatment *n* = 4; Asc *n* = 3). Statistical significance was calculated using one-way ANOVA followed by Bonferroni’s post hoc tests and unpaired *t*-test for H_2_O_2_ (in **D**, **F**), using two-way ANOVA followed by Bonferroni’s post hoc tests (in **E**), *significant compared to the solvent control, ^#^significant compared to TM single treatment. (*p* ≤ 0.05 was considered significant). SC, solvent control (N2-medium); OSGIN1, oxidative stress induced growth inhibitor 1; HMOX1, heme oxygenase 1; NQO1, NAD(P)H dehydrogenase quinone 1; GCLM, glutamate-cystein ligase modifier subunit; ME1, malic enzyme 1; HDLBP, high-density lipoprotein binding protein; H_2_O_2_, hydrogen peroxide; Asc, ascorbic acid

Since oligodendrocyte differentiation was the only analyzed neurodevelopmental endpoint affected by TM, we performed microarray analyses of hNPCs differentiated for 60 h under exposure to either 1 mg/mL TM or vehicle (differentiation medium without TM; Fig. 5) to help identify the underlying MoA. Exposure to 1 mg/mL TM during the 60 h of neurosphere differentiation significantly (*p* ≤ 0.05, fold change ≥ 2) regulated the expression of 22 genes compared to the respective controls (Fig. 5A, C; Suppl. Tab. S5). Here, an increased cutoff stringency is necessary for identifying oligodendrocyte-specific MoAs, since oligodendrocytes represent only approximately 5% of the total differentiated hNPC culture. A gene set enrichment analysis revealed only 12 GO terms, of which more than half (58%) were associated with cell death/apoptosis, while 25% and 17% were related to cell signaling and other biological processes, respectively (Fig. 5A). Looking at the GO terms associated with cell death, a set of 5 genes (oxidative stress induced growth inhibitor 1, *OSGIN1*; heme oxygenase 1, *HMOX1*; NAD(P)H dehydrogenase quinone 1, *NQO1*; glutamate-cystein ligase modifier subunit, *GCLM*; malic enzyme 1; *ME1*) related to oxidative stress were overrepresented (Suppl. Fig. S3). In accordance with that, STRING gene–gene interaction network analysis based on the 22 significantly regulated genes revealed a high correlation of the 5 genes involved in oxidative stress response (Fig. 5B). In addition to the 5 differentially expressed genes related to oxidative stress (red dots, Fig. 5C), we identified 1 gene (high-density lipoprotein (HDL) binding protein, *HDLBP*) to be involved in lipid/cholesterol metabolism (green dot, Fig. 5C), a process necessary for myelination and thereby for proper oligodendrogenesis (Berghoff et al. 2016). *HDLBP* binds HDL, such as cholesterol, thereby regulating excess cholesterol levels in cells. An *HDLBP* upregulation (FC 2.3) caused by TM may lead to excessive HDL binding, thus reducing the availability of HDL for myelin formation, which in turn disturbs oligodendrogenesis (Li et al. 2020a; Nelissen et al. 2012). Next to *NQO1* and *HMOX1*, 3 additional DEX genes (mitogen-activated protein kinase 1, *MAP2K1*; SRY-Box transcription factor 5, *SOX5*; Wnt family member 3, *WNT3*) are associated with oligodendrogenesis (blue dots, Fig. 5C). Downregulation of Wnt signaling, Sox5 transcriptional activity, and MAP2K1-dependend ERK1/2 activation are necessary for oligodendrocyte precursor (OPC) proliferation and differentiation (Shimizu et al. 2005; Langseth et al. 2010), the maintenance of OPCs in an undifferentiated state (Baroti et al. 2016), and the synthesis of myelin or remyelination (Jeffries et al. 2016), respectively. In case of *SOX5*, the observed upregulation (FC 2.1) might lead to a continuous sojourn of OPCs in the immature state (Baroti et al. 2016), thereby decelerating oligodendrogenesis and decreasing oligodendrocyte numbers. Since we observed *WNT3* to be downregulated (FC 2.0) and *MAP2K1* to be upregulated (FC 2.1) upon TM treatment, we hypothesize a compensatory mechanism in response to the reduced oligodendrocyte differentiation. These observations have to be more intensively examined in the future.

Altogether, based on these transcriptomic findings, we hypothesize that oxidative stress plays a significant role in the oligodendrocyte reduction observed after treatment with TM. Oxidative stress is defined as an imbalance between the production of ROS and the antioxidant capacity of the cell. Measurements of ROS accumulation in differentiated hNPCs exposed to 1 mg/mL TM (60 h) revealed significantly induced ROS levels up to 158.5 ± 14.4% of untreated controls (Fig. 5D). As expected, the phenotypically unobtrusive concentration of 0.01 mg/mL TM did not cause oxidative stress (Fig. 5D). In order to confirm that TM toxicity on hNPCs is ROS dependent, hNPCs were co-exposed to TM (1 mg/mL) and the antioxidant ascorbic acid (Asc; 100 µM; Fig. 5E). Asc significantly antagonized the TM-dependent inhibition of oligodendrocyte differentiation to 97.1 ± 6.2% of control, while Asc alone did not have a significant impact (Fig. 5E). Hence, these observations support the transcriptomic data and the endophenotype of decreased hNPC oligodendrocyte differentiation. However, at this point, we cannot exclude that our findings on the transcriptomic level, the ROS induction, and the Asc rescue can be attributed to other cells but oligodendrocytes in our mixed culture system. However, concerning TM exposure, oligodendrocytes are the most sensitive cell type (Fig. 4B).

Our hypothesis of ROS-induced oligodendrocyte toxicity is supported by previous clinical and cell biological investigations. Specifically developing human oligodendrocytes, i.e., pre-oligodendrocytes exert a high susceptibility towards ROS (Volpe et al. 2011; van Tilborg et al. 2016). The pathognomonic relevance of ROS for impaired oligodendrogenesis is expressed in white matter injuries (WMI) occurring in premature infants. Here, free radical attack on pre-oligodendrocytes was identified as one of the major pathomechanisms responsible for the disease causing either oligodendrocyte death or oligodendrocytes with impaired cellular functions, e.g., reduced differentiation or myelination (Volpe et al. 2011). The ROS sensitivity of pre-oligodendrocytes has multiple causes. On the one hand, it is due to their low glutathione (GSH)- and superoxide dismutase-dependent antioxidative defense, their increased expression of ROS-producing 12/15 lipoxygenase (Folkerth et al. 2004; Haynes and Van Leyen 2013), and their enormous intracellular stores of iron, which is the largest in the brain (Juurlink 1997; Back et al. 1998; Marinelli et al. 2016). Iron ions in presence of hydrogen peroxide and superoxide anions catalyze formation of the highly reactive hydroxyl radical by the Haber–Weiss reaction (Haber and Weiss 1934). Furthermore, oligodendrocytes synthesize more than threefold their own weight of myelin (Norton and Poduslo 1973) and facilitate membrane production of up to 100 × the weight of their cell bodies per day (Ludwin 1997; McTigue and Tripathi 2008; Bradl and Lassmann 2010). The myelin sheath is characterized by a high content of lipids (70–85%; Williams and Deber 1993; Poitelon et al. 2020) illustrating the high vulnerability of oligodendrocytes towards ROS-initiated lipid peroxidation leading to cell death or malfunction of oligodendrocytes (Haq et al. 2003; Bezine et al. 2017).

In general, TM was suggested to possess antioxidative and neuroprotective properties (reviewed in Zhan et al. 2016 and Heese 2020; Xian et al. 2016) by recommending a daily therapeutic intake of 10 g/person (Chinese Pharmacopoeia Commission 2020). However, most studies deal with single TM components and/or are based on in vivo studies in adult animals (Shuchang et al. 2008; Park et al. 2015; Liu et al. 2018b, 2020; Jiang et al. 2020). So far, only a few human cell-based studies reported altered ROS responses after exposure to gastrodin, one of the main components of TM. Here, gastrodin acted as an antioxidant on human retinal endothelial cells (Zhang et al. 2018), while it induced ROS-associated cytotoxicity in human glioblastoma cells (Liang et al. 2017). In vivo, TM exerted no obvious adverse effects, e.g., malformations, on prenatally treated rat fetuses by testing doses up to 6.16 g/kg (Yuan et al. 2013). However, WMI, especially more subtle forms like diffuse WMI or punctate white matter lesions (van Tilborg et al. 2016), were not investigated in these studies. Hence, in vivo effects cannot be excluded by the currently available data. Another possibility is that due to species differences between rodents and humans, an animal experiment could deliver a false-negative result. Including time-matched rat neurospheres into our studies (Workman et al. 2013; Baumann et al. 2014), we observed no TM-induced oligodendrocyte toxicity in differentiating rat NPCs, which is in strong contrast to the TM-induced disturbance of oligodendrocyte differentiation we detected in hNPCs (Fig. 4B, D, Fig. 5F). We previously demonstrated that human NPCs are more sensitive towards arsenite-induced oxidative stress than rat NPCs due to their approximately three times lower basal expression of GSH-dependent and GSH-independent antioxidative defense-related genes and their lower GSH content (Masjosthusmann et al. 2019). Regarding oxidative stress, higher protection against ROS-mediated toxicity in rodents compared to humans was reported for arsenite-exposed embryonic mouse brains (Allan et al. 2015), thalidomide-mediated toxicity in rat and rabbit whole embryo cultures (Hansen et al. 1999), embryonic fibroblasts in vitro, and adult heart tissue in vivo (Janssen et al. 1993; Knobloch et al. 2008). It is increasingly recognized that the physiology of laboratory animals often differs from human physiology (Knight 2007; Leist and Hartung 2013). For example, Olson et al. (2000) demonstrated that rodents identified only 43% of 150 pharmaceuticals known to be toxic in humans. This is especially true for cellular aspects concerning the brain as central nervous system (CNS) drug development has been plagued by a failure to translate effective therapies from the lab to the clinic (Dragunow 2020).

### Putative AOP network for the disruption of oligodendrocyte development

**Fig. 6:**
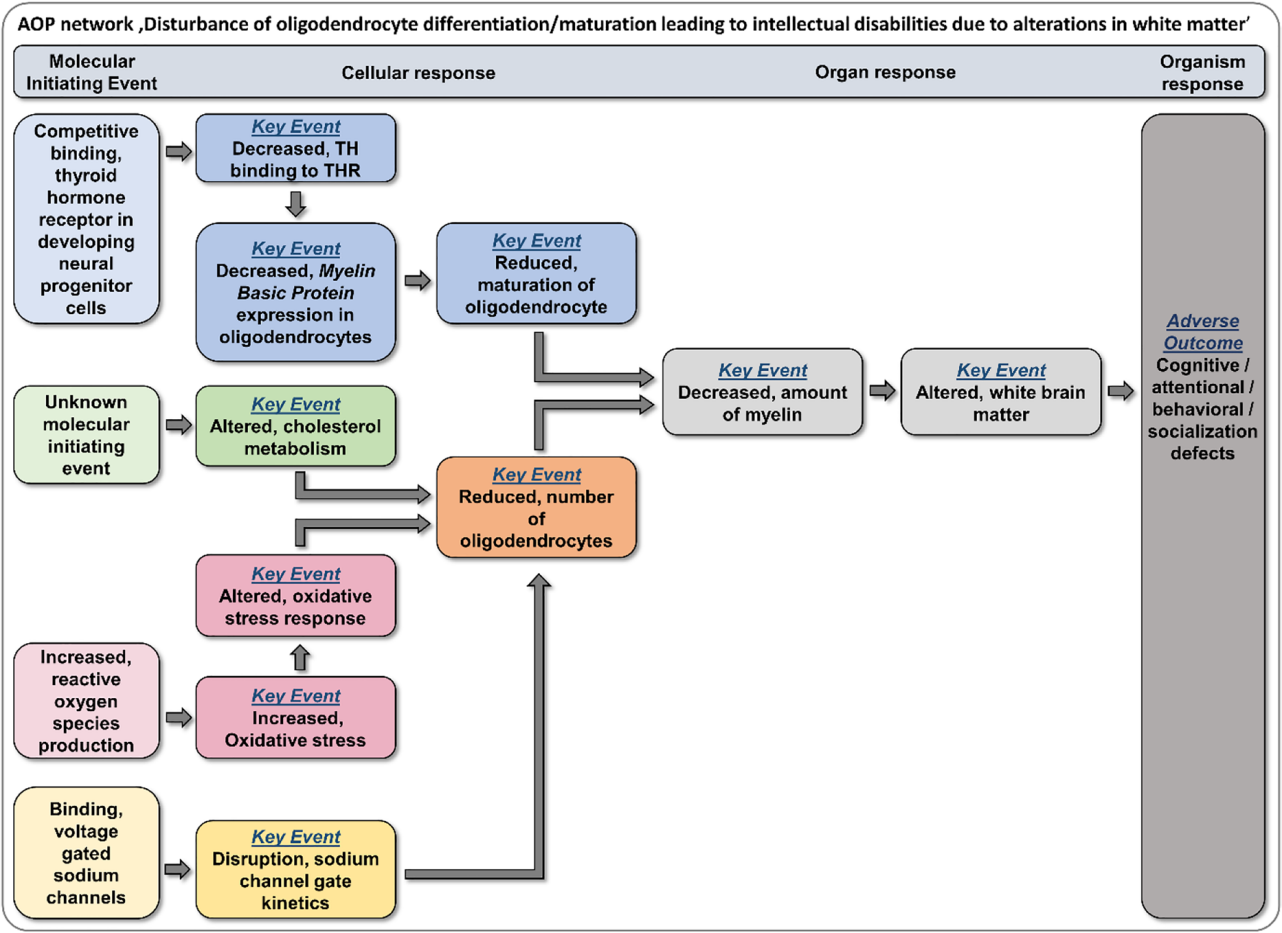
Putative AOP network for impaired oligodendrocyte development. The previously established oligodendrocyte development AOP network (Klose et al. 2021b) is enlarged by the MIE “increased reactive oxygen species production” and KEs regarding oxidative stress. In addition, the MIE and KE regarding sodium channels and their impact on oligodendrocyte differentiation is included (Hernández-Jerez et al. 2021). This illustration represents a qualitative network and does not include dashed key event relationships

Combining the endophenotypes of the “Neurosphere Assay” with microarray analysis, we identified one putative MoA how TM might interfere with oligodendrocyte development. This MoA involves the dysregulation of a gene expression network involved in oxidative stress. Since especially pre-oligodendrocytes are highly susceptible towards oxidative stress, TM exposure might reduce the number of differentiated oligodendrocytes from hNPC by increasing intracellular ROS levels. This observation leads us to the implementation of a further stressor-dependent AOP with the MIE “increased reactive oxygen species production” along with KEs connected to oxidative stress into our recently published stressor-based AOP network (Klose et al. 2021b; Fig. 6). Increased ROS production (KE257; https://aopwiki.org/) as a MIE was previously observed in response to oxidative stressors (e.g., radiation, metals and organics) and is currently under development within the AOP-Wiki (Song et al. 2020b; https://aopwiki.org/; #238, #299, #311, #327–330, #386–387). One AOP (#17; http://aopwiki.org) already in the AOP-Wiki describes a linkage between oxidative stress and impairment in learning and memory, yet independent of oligodendrocytes. The AOP network around the reduced number of oligodendrocytes will therefore link to AOP #17 via the KE “Oxidative stress.” In addition, the KE “Reduced number of oligodendrocytes” connects upstream to KEs related to binding of compounds to voltage-gated sodium channels in a currently published stressor (deltamtherin)-dependent AOP network (Hernández-Jerez et al. 2021). Both the KE “Altered cholesterol metabolism” and KE “altered oxidative stress response” in our AOP network impair oligodendrocyte differentiation, thus reducing their numbers and potentially impairing myelin production. In addition, also effects on oligodendrocyte maturation (KE “Reduced maturation of oligodendrocytes”) can in the end result in reduced brain myelin. Lower myelin causes alterations in the white matter leading to adverse outcomes such as cognitive, attentional, behavioral, and/or social deficits (Back et al. 2001; Gika et al. 2010; La Piana et al. 2015; Berghoff et al. 2017). Obviously, more compounds acting via these MoA need to be identified in order to strengthen this hypothetical AOP. In addition, KE relationships have to be experimentally established.

## Summary and conclusions

In summary, we tested two selected CHMs, LGT and TM, for their potential to induce neurodevelopmental toxicity by application of the human cell-based in vitro “Neurosphere Assay,” which is part of the EFSA DNT in vitro battery (Masjosthusmann et al. 2020). Both CHM caused previously observed DNT endophenotypes, i.e., disturbed radial glia migration and impaired oligodendrocyte differentiation. Transcriptome analyses added molecular data to the cellular phenotypic observations. The LGT-induced migration endophenotype was rescued by laminin suggesting disturbances in cell adhesion to laminin as the MoA, which maps our new observations to an already published AOP on “Disrupted laminin-beta1-integrin interaction leading to developmental neurotoxicity” (Bal-Price et al. 2016; Barenys et al. 2017a). The molecular data on TM exposure unraveled a novel stressor-dependent AOP on disturbed oligodendrocyte differentiation upon ROS accumulation along with oxidative stress that was added to the previously published AOP network on impaired oligodendrocyte development (Hernández-Jerez et al. 2021; Klose et al. 2021b). Hence, CHM are a valuable example for compounds in broad public use with insufficient hazard characterization concerning DNT. By testing whole CHM extracts, which are mixtures of natural substances, we addressed a relevant “real-life” exposure scenario.

This study demonstrates the power of combining phenotypic with transcriptomic analyses for better understanding MoA and applying or building AOPs for DNT. Especially the multi-cellularity of the “Neurosphere Assay,” comprising NPCs, radial glia, neurons, astrocytes, and oligodendrocytes, makes this test method an effective instrument for multiple MoA discovery due to its broad applicability domain (Masjosthusmann et al. 2020). Increasing the number of test methods to cover more KEs, such as neuronal network formation, synaptogenesis, and radial-/astro-/microglia formation/function or activation, will eventually lead to larger DNT-AOP networks. Besides CHM, for most substances in our direct environment, we lack information concerning their effects on neurodevelopmental endpoints (Tsuji and Crofton 2012; Fritsche et al. 2018b; Sachana et al. 2019). This concerns single compounds as well as mixtures. In the future, in vitro and in vivo toxicokinetics need to be considered for moving from hazard to risk assessment for a broad variety of substance classes to best protect the brains of our future generations.

## Supplementary Information

Below is the link to the electronic supplementary material.

Supplementary file1 (PDF 510 KB)

Supplementary file2 (WMV 9498 KB)

Supplementary file3 (WMV 10176 KB)

Supplementary file4 (WMV 6748 KB)

Supplementary file5 (WMV 4648 KB)

## Data Availability

The dataset generated during and/or analyzed during the current study are available from the corresponding author on reasonable request.
