# Supplementary material for: Application of the adverse outcome pathway concept for investigating developmental neurotoxicity potential of Chinese herbal medicines by using human neural progenitor cells in vitro
Source: Cell Biol Toxicol. 2022 Jun 15;39(1):319–43. doi: 10.1007/s10565-022-09730-4 (PMC10042984; doi:10.1007/s10565-022-09730-4)
Supplement: Supplementary file 1 — Supplementary file1 (PDF 510 KB) [file 10565_2022_9730_MOESM1_ESM.pdf]

Supplementary Material

Application of the Adverse Outcome Pathway concept for investigating developmental neurotoxicity potential of Chinese Herbal Medicines by using human neural progenitor cells *in vitro*

Jördis Klose[1]*, Lu Li[2,3,4,9]*, Melanie Pahl[1], Farina Bendt[1], Ulrike Hübenthal[1], Christian Jüngst[5], Patrick Petzsch[6], Astrid Schauss[5], Karl Köhrer[6], Ping Chung Leung[4], Chi Chiu Wang[3,7,8], Katharina Koch[1], Julia Tigges[1], Xiaohui Fan[2,9]†, Ellen Fritsche[1,8,10]†

[1] IUF-Leibniz Research Institute for Environmental Medicine, Auf'm Hennekamp 50, 40225 Duesseldorf, NRW, Germany

[2] College of Pharmaceutical Sciences, Zhejiang University, Hangzhou, China

[3] Department of Obstetrics & Gynaecology; Li Ka Shing Institute of Health Sciences; School of Biomedical Sciences, The Chinese University of Hong Kong, Shatin, N.T., Hong Kong

[4] Institute of Chinese Medicine, The Chinese University of Hong Kong, Shatin, N.T., Hong Kong

[5] CECAD Imaging Facility, CECAD Forschungszentrum Cologne, Joseph-Stelzmann-Str. 26, 50931 Cologne, NRW, Germany

[6] Biological and Medical Research Centre (BMFZ), Medical Faculty, Heinrich-Heine-University, Universitätsstraße 1, 40225 Duesseldorf, NRW, Germany

[7] Joint Laboratory in Reproductive Medicine, The Chinese University of Hong Kong and Sichuan University, China

[8] College of Basic Medical Sciences, Zhejiang Chinese Medical University, Hangzhou, China

[9] Innovation Center in Zhejiang University, State Key Laboratory of Component-Based Chinese Medicine, Hangzhou, China

[10] Medical Faculty, Heinrich-Heine-University, Universitätsstraße 1, 40225 Duesseldorf, NRW, Germany

Correspondence: Prof. Dr. Ellen Fritsche
Phone: +49 (0) 211 3389 217
E-Mail: ellen.fritsche@uni-duesseldorf.de

* † authors contributed equally

Supplementary Table S1: LD_{50} values of the Tian Ma and Lei Gong Teng extracts as well as of single components.

CHM extract / components	Administration	Species	LD_{50}	Reference
**TM (whole extract)**	intraperitoneally	mouse (male)	61.4 g/kg	(Shen and Chang 1963)
	intraperitoneally	mouse (female)	51.4 g/kg	
	intravenously	mouse	39.8 g/kg	(Huang 1985)
Vanillic alcohol	intraperitoneally	mouse	891.3 mg/kg	(Liu et al. 1974)
Vanillin	intraperitoneally	mouse	946.0 mg/kg	
**LGT (whole extract)**	*intragastrically*	mouse	112.0 g/kg	(Zhen et al. 1982)
	intraperitoneally	mouse	50.5 g/kg	
Total glycoside	*intragastrically*	mouse	159.7 mg/kg	(Zhen 1983)
	intraperitoneally	mouse	93.9 mg/kg	
Root bark decoction	*intragastrically*	rat (female)	21.6 g/kg	(Zhang et al. 1983)
Triptolide	intraperitoneally	mouse	1.41 mg/kg	(Zhang et al. 1980)
	intravenously	mouse	0.80 mg/kg	(Zhen et al. 1994)
	intraperitoneally	mouse	0.90 mg/kg	
	intravenously	dog	160.0 µg/kg	

Supplementary Table S2: Ingredients included in extractions of Lei Gong Teng and Tian Ma. Research was based on records in Chinese Pharmacopoeia and/or literatures. Divided in respective parts of the plant.

CHM	Part of plant	Main components/compounds
**Tian Ma (Gastrodia elata Blume)**	**Rhizoma**	Gastrodin
		Gastrodioside
		p-hydroxybenzyl alcohol
		p-hydroxybenzaldehyd
		4-hydroxybenzyl methyl ether
		4- (4'-hydroxybenzyloxy)- benzyl methyl ether
		Bis-(4-hydroxybenzyloxy) ether
		Vanillyl alcohol
		Citric acid
		Methyl citrate
		Succinic acid
		Palmitic acid
		β-sitosterol
		Daucosterol
		Sucrose
	**Stem/root /rootstock**	Gastrodia antifungal protein
		Chitinase
		β-1,3-glucanase
		Gastrodia elata polysaccharide
		Trace elements: Fe / F / Mn / Zn / Sr / I / Cu
	**Fresh plant**	Gastrodin
		p-hydroxybenzyl alcohol
		p-hydroxybenzaldehyd
		3,4-dihydroxybenzyloxybenzaldehyde
		4,4-dihydroxydiphenyl methane
		p-hydroxybenzyl ethyl ether
		4,4-dihydroxydibenzyl ether
		4-ethoxymethylphenyl-4'-hydroxybenzyl ether
		Tris-[4-(β-D-glucopyranosyloxy)-benzyl] citrate, Parishin
		4-ethoxymethylphenol

CHM	Part of plant	Main components/compounds	
Lei Gong Teng (Common Threewingnut Root)	Root	Wilfordine	Celacinnine
		Wilforine	Celafurine
		Wilforgine	Wilforlide A, B
		Wilfortrine	Triptonoterpenol
		Wilforidine	16-hydroxytriptolide
		Wifornine, Euonine	Triptolide
		Wilforzine	Epitriptriolide
		Neowilforine	Tripterifordine
		1-desacetyl wilfordine	Antriptolactone
		1-desacetyl wilfortrine	Tripterygic acid
		2-debenzoyl-2-nicotinoyl wilforine	Orthosphenic acid
		Isowilfordine	β-sitosterol
		Wilforcidine	Daucosterol
		Celagbenzine	
	Xylem of root	Triptoterpenoid lactone A	2α,3α,24-trihy- droxy-12-ursene-28-oic acid
		Wilforlide A, B	Tripterygone
		Celastrol or Tripterine	Tripchlorolide
		3β-22α-dihydroxy-12-oleanen-29-oic acid	Triptriolide
		3,24-dioxo-friedelan-29-oic acid	Linolenic acid
		3-epikatonic acid	8,9-octadecadienoic acid
		Salaspermic acid	Oleic acid
		Triptotriterpenic acid A, B, C	9-hexadecenoic acid
		Orthosphenic acid	Palmitic acid
		3β,22β-dihydroxy-12-oleanen-29-oic acid	Stearic acid
	Skin of root	Wilfordine	Triptonoterpene methyl ether
		Wilforine	Triptolidenol
		Wilforgine	Hypolide
		Wifornine, Euonine	Hypolide methylether
		Wifornine	Isoneotriptophenolide
		Wilforjing	Triptriolide
		Wilfortrine	Triptotetraolid
		Triptonolide	Isotr iptetraolide
		Triptonide	Wilforonide
		Triptolide	Wilforlide A
		Tripdiolide	Glut-5-en-3β, 28-diol
		Trip-tophenolide	Ursan-3β, 5α-diol
		Triptophenolide methyl ether	Polpunonic acid, Populnonic acid, Maytenonic acid
		Neotriptophenolide	Triptodihy-droxy acid methyl ester
		Triptonoterpene, 14-hydroxy-abieta-8, 11, 13-trien-3-one	

Supplementary Material and Method

Proliferation

To evaluate the impact of LGT and TM on hNPC proliferation, both CHMs were tested in serial dilutions (1:10) with 7 concentrations as well as respective solvent (proliferation medium) and plated in five replicate wells per condition in a 96-well U-bottom plate. Per well, one sphere with a defined size of 0.3 mm was plated in 100 µL of proliferation medium containing the respective CHM concentration and incubated for 72 h under standard culture conditions (37°C and 5% CO_2). The proliferation by area was assessed as slope of the increase in sphere size up to 3 days (0 h, 24 h, 48 h and 72 h) measured by brightfield microscopy and using high content imaging (Cellomics Scan software, Version 6.6.0; Thermo Fisher Scientific). Proliferation by Bromdesoxyuridin (BrdU) was analyzed after 72 h of exposure via a luminescence-based BrdU assay (Roche) as previously published in Nimtz et al. (2019). In parallel, viability and cytotoxicity assays were performed. Therefore, hNPC viability was assessed as mitochondrial activity by using an Alamar blue assay (CellTiter-Blue assay (CTB); Promega) in the last two hours of the 72 h exposure. Cytotoxicity of treated hNPCs was detected by measuring LDH in the supernatant, which was removed before the CTB assay was started by using the CytoTox-ONE membrane integrity assay (Promega).

Supplementary Figure S1

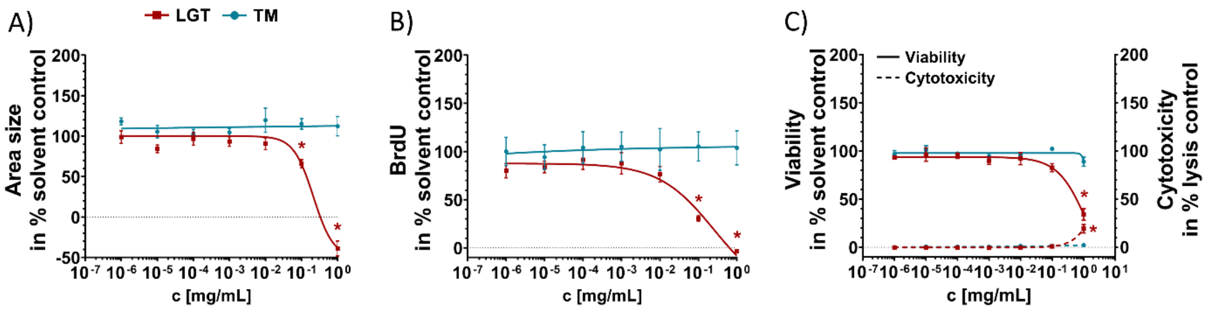

Fig. S1: Influence of LGT and TM on proliferating hNPCs. Spheres with a defined size of 0.3 mm were plated in 96-well U-bottom plates and exposed to increasing CHM concentrations over 72 h. Proliferation was studied by measuring the increase of sphere area (**A**) and by quantifying BrdU incorporation into the DNA (**B**). In parallel, viability and cytotoxicity (**C**) were assessed by performing Alamar Blue (viability) and LDH assay (cytotoxicity). At least three independent experiments with 5 technical replicates were performed and presented as mean ± SEM. Statistical significance was calculated using OneWay ANOVA followed by Bonferroni's post-hoc tests ($p \leq 0.05$ was considered significant). BrdU = bromodeoxyuridine.

Supplementary Figure S2

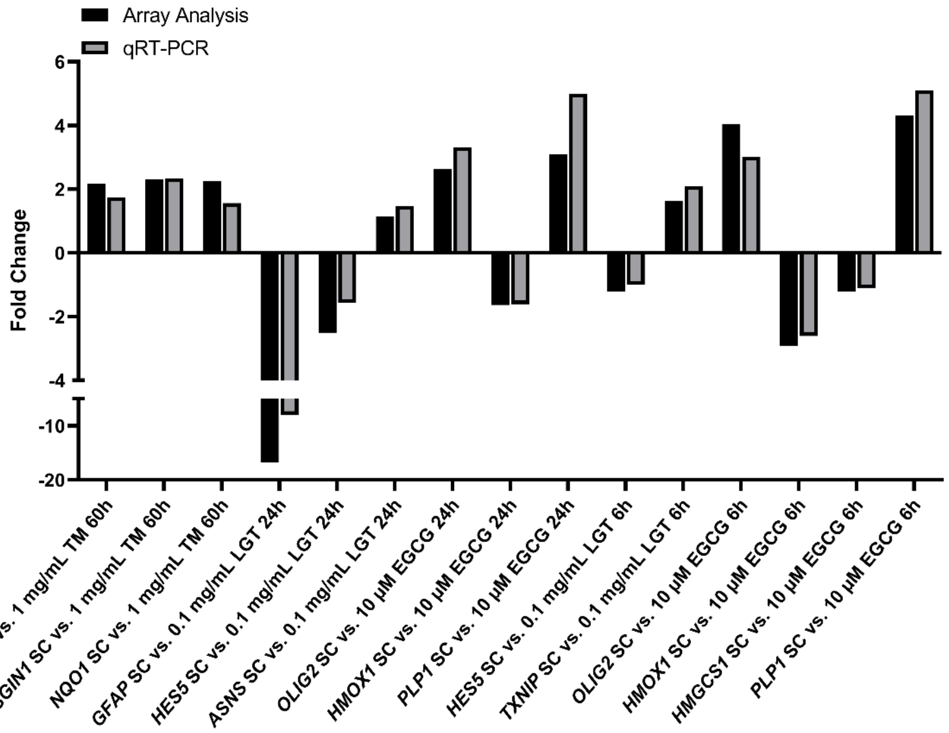

Fig. S2: Quantitative RT PCR validation of array analysis. Validation of microarray data was performed with quantitative real-time polymerase chain reactions (qRT-PCR) analysis of a set of ten genes. The fold changes of qRT-PCR (grey bars) were compared to the fold changes of the array analysis (black bars). A total of 500 ng RNA from microarray samples were transcribed into cDNA (complementary DNA). qRT-PCR was performed using the QuantiFast SYBR Green PCR Kit and a Rotor Gene Q Cycler (Qiagen). Analysis was performed using the software Rotor-Gene Q Series version 2.3.4. SC = solvent control; *HMOX1* = heme oxygenase 1; *OSGIN1* = oxidative stress induced growth inhibitor 1; *NQO1* = NAD(P)H dehydrogenase quinone 1; *GFAP* = Glial fibrillary acidic protein; *HES5* = Hes Family BHLH Transcription Factor 5; *ASNS* = Asparagine Synthetase; *OLIG2* = Oligodendrocyte Transcription Factor 2; *PLP1* = Proteolipid Protein 1; *TXNIP* = *Thioredoxin Interacting Protein*; *HMGCS1* = Hydroxymethylglutaryl-CoA synthase 1.

Supplementary Table S3: Set of 10 genes used for microarray validation. Forward and reverse primer sequences are listed in 5'-3'. *HMOX1* = heme oxygenase 1; *OSGIN1* = oxidative stress induced growth inhibitor 1; *NQO1* = NAD(P)H dehydrogenase quinone 1; *GFAP* = Glial fibrillary acidic protein; *HES5* = Hes Family BHLH Transcription Factor 5; *ASNS* = Asparagine Synthetase; *OLIG2* = Oligodendrocyte Transcription Factor 2; *PLP1* = Proteolipid Protein 1; *TXNIP* = *Thioredoxin Interacting Protein*; *HMGCS1* = Hydroxymethylglutaryl-CoA synthase 1.

Primer	Forward sequence	Reverse sequence
BETA-ACTIN	5'-CAGGAAGTCCCTTGCCATCC-3'	5'-ACCAAAAGCCTTCATACATCTCA-3'
HMOX1	5'-GCCATGAACTTTGTCCGGTG-3'	5'-GGATGTGCTTTTCGTTGGGG-3'
OSGIN	5'-TCATCATTGTGGGTAACGGC-3'	5'-CTTCGTGTAGGGTGTGTAGC-3'
NQO1	5'-TATCCTGCCGAGTCTGTTCT-3'	5'-TGCAGGGGGAACTGGAATA-3'
GFAP	5'-CACTGTGAGGCAGAAGCTC-3'	5'-CCTCCAGCGACTCAATCTTC-3'
HES5	5'-TCCCTGCCGTTTTAGGACAA-3'	5'-TACGGGCCCTGAAGAAAGTC-3'
ASNS	5'-CTGCACGCCCTCTATGACA-3'	5'-TAAAAGGCAGCCAATCCTTCT-3'
OLIG2	5'-CCGATGACCTTTTTCTGCCG-3'	5'-CCACTGCCTCCTAGCTTGTC-3'
PLP1	5'-TTGGCGACTACAAGACCACC-3'	5'-GGGAAGGCAATAGACTGGCA-3'
TXNIP	5'-CCTGAAAAGGTGTACGGCAG-3'	5'-TCTCATTCTCACCTGTTGGC-3'
HMGCS1	5'-TATTCCAAGCCCTGCCAAGA-3'	5'-TCCAACTGTTCCCATACCCC-3'

Supplementary Table S4: Gene regulation of migrating neurospheres exposed to 0.01, 0.1 mg/mL Lei Gong Teng (LGT) and 10 µM Epigallocatechin gallate (EGCG) for 6 h (#22) and 24 h (#18). The fold changes of genes regulated by both compounds were identified by array analysis. Upregulated genes are shown as positive values and downregulated genes are shown as negative fold changes (-).

Timepoint	Gene	regulated by 0.01 LGT	regulated by 0.1 LGT	regulated by 10 µM EGCG
6 hours	FAXDC2	1.5	1.9	1.9
	ACSS3	1.6	2.1	-2.4
	MT1H	-1.9	1.7	-2.0
	MT1G	-2.1	1.6	-2.4
	ZNF567	1.6	1.8	1.5
	HIST1H2BM	-2.0	-1.5	2.0
	ST7	2.2	2.0	1.8
	THOC1	2.0	2.0	1.6
	NIN	2.0	1.8	1.9
	TLR3	1.9	2.2	2.1
	THAP2	1.8	1.8	1.7
	KIAA1715	1.6	1.9	1.5
	SESN3	1.6	2.0	2.3
	GLCCI1	1.6	1.9	5.4
	CDK19	1.6	1.7	1.6
	ZIK1	1.6	1.6	1.6
	NBR1	1.6	1.8	1.5
	RASGEF1B	1.5	1.8	2.8
	HIST3H2BB	-1.5	-1.5	1.6
	SPRY4	-1.6	-2.0	-1.7
	CDON	1.8	2.0	5.6
	TCP11L2	1.6	1.7	1.5
24 hours	SNRPD3	1.6	1.7	1.5
	MT1H	-1.9	-2.1	-4.0
	MT1X	-1.6	-2.8	-4.6
	MT1B|MT1CP	-1.9	-2.3	-4.2
	PLEC	-1.7	-2.8	-2.1
	OSTM1	1.5	2.0	1.5
	ICE2	1.6	1.7	1.7
	ATAD2B	1.6	1.6	1.5
	LGALS1	-1.6	-2.1	-1.9
	IFITM3	-1.6	-1.8	-12.8
	SNAP29	1.6	1.6	1.5
	RBM48	1.5	1.5	1.8
	MT1A	-1.7	-2.3	-3.3
	MT2A	-1.7	-2.4	-3.1
	MT1L	-1.6	-2.1	-4.0
	MAPK14	1.5	1.5	1.7
	PAPOLG	1.7	1.7	1.7
	RGCC	1.6	1.5	2.4

Supplementary Table S5: Gene regulation of migrating/differentiating neurospheres exposed to 1 mg/mL Tian Ma (TM) for 60 h (#22). The fold changes of genes regulated by both compounds were identified by array analysis. Upregulated genes are shown as positive values and downregulated genes are shown as negative fold changes (-).

Timepoint	Gene	regulated by 1 mg/mL TM
60 hours	PTPN13	3.1
	ALG6	2.4
	GNGT1	-2.3
	OSGIN1	2.3
	GCLM	2.3
	PLCZ1	-2.3
	SKAP2	2.3
	HDLBP	2.3
	NQO1	2.3
	HMOX1	2.2
	KRTAP2-3	-2.2
	DFNA5	2.2
	CLIP4	2.2
	TMEM99	2.1
	SMC6	-2.1
	MAP2K1	2.1
	ME1	2.1
	SOX5	2.1
	SNX5	2.1
	F2RL2	2.1
	XKRY	-2.1
	WNT3	-2.0

Supplementary Figure S3

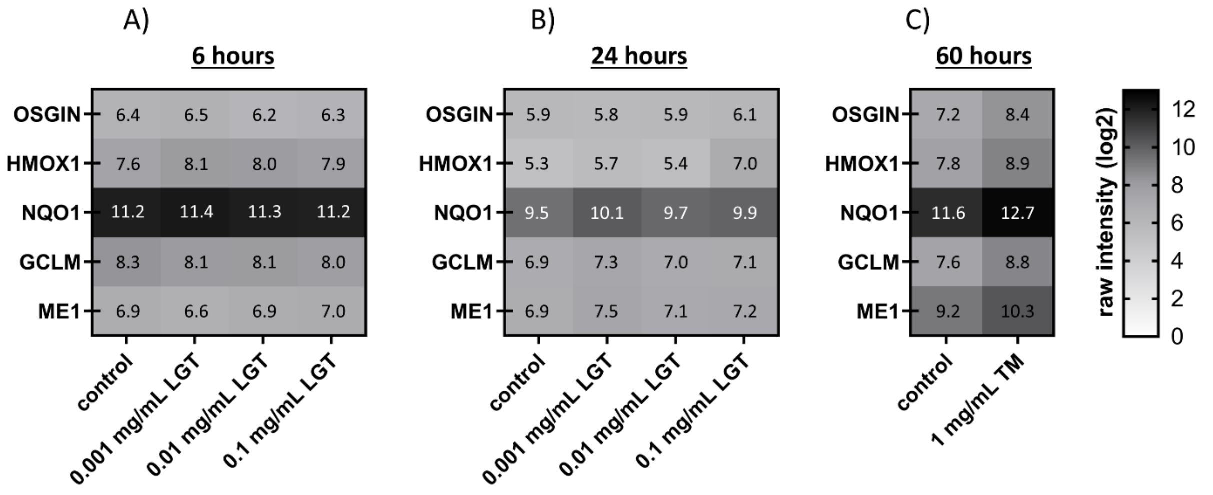

Fig. S3: Transcriptomic profiling related to oxidative stress of differentiated hNPCs treated with LGT and TM. Gene expression profile between hNPCs exposed to LGT (0.001, 0.01, 0.1 mg/mL) and untreated hNPCs over 6 h (**A**) and 24 h (**B**) of migration/differentiation, as well as 60 h (**C**) migrated/differentiated hNPCs exposed to 1 mg/mL TM. Values are shown in absolute signal intensity (log2) of oxidative stress associated genes (oxidative stress induced growth inhibitor 1, *OSGIN1*; heme oxygenase 1, *HMOX1*; NAD(P)H dehydrogenase quinone 1, *NQO1*; glutamate-cysteine ligase modifier subunit, *GCLM*; malic enzyme 1; *ME1)*
